# Extracellular matrix-specific Caveolin-1 phosphorylation on tyrosine 14 is linked to augmented melanoma metastasis but not tumorigenesis

**DOI:** 10.18632/oncotarget.9738

**Published:** 2016-05-31

**Authors:** Rina Ortiz, Jorge Díaz, Natalia Díaz, Lorena Lobos-Gonzalez, Areli Cárdenas, Pamela Contreras, María Inés Díaz, Ellen Otte, Justin Cooper-White, Vicente Torres, Lisette Leyton, Andrew F.G. Quest

**Affiliations:** ^1^ Center for Molecular Studies of the Cell (CEMC), Advanced Center for Chronic Diseases (ACCDiS), Faculty of Medicine, Universidad de Chile, Santiago, Chile; ^2^ Laboratory of Cellular Communication, Program of Cell and Molecular Biology, Institute of Biomedical Sciences (ICBM), Faculty of Medicine, Universidad de Chile, Santiago, Chile; ^3^ Universidad Bernardo O Higgins, Facultad de Salud, Departamento de Ciencias Químicas y Biológicas, Santiago, Chile; ^4^ Institute for Research in Dental Sciences, Faculty of Dentistry, Universidad de Chile, Santiago, Chile; ^5^ Andes Biotechnologies SA, Ñuñoa, Santiago, Chile; ^6^ Fundación Ciencia & Vida, Ñuñoa, Santiago, Chile; ^7^ Biomedical Neuroscience Institute (BNI) Santiago, Chile; ^8^ Australian Institute for Bioengineering & Nanotechnology, The University of Queensland, St. Lucia, Queensland, Australia

**Keywords:** Caveolin-1, cancer, dual role, migration, invasion

## Abstract

Caveolin-1 (CAV1) is a scaffolding protein that plays a dual role in cancer. In advanced stages of this disease, CAV1 expression in tumor cells is associated with enhanced metastatic potential, while, at earlier stages, CAV1 functions as a tumor suppressor. We recently implicated CAV1 phosphorylation on tyrosine 14 (Y14) in CAV1-enhanced cell migration. However, the contribution of this modification to the dual role of CAV1 in cancer remained unexplored. Here, we used *in vitro* [2D and transendothelial cell migration (TEM), invasion] and *in vivo* (metastasis) assays, as well as genetic and biochemical approaches to address this question in B16F10 murine melanoma cells. CAV1 promoted directional migration on fibronectin or laminin, two abundant lung extracellular matrix (ECM) components, which correlated with enhanced Y14 phosphorylation during spreading. Moreover, CAV1-driven migration, invasion, TEM and metastasis were ablated by expression of the phosphorylation null CAV1(Y14F), but not the phosphorylation mimicking CAV1(Y14E) mutation. Finally, CAV1-enhanced focal adhesion dynamics and surface expression of beta1 integrin were required for CAV1-driven TEM. Importantly, CAV1 function as a tumor suppressor in tumor formation assays was not altered by the Y14F mutation. In conclusion, our results provide critical insight to the mechanisms of CAV1 action during cancer development. Specific ECM-integrin interactions and Y14 phosphorylation are required for CAV1-enhanced melanoma cell migration, invasion and metastasis to the lung. Because Y14F mutation diminishes metastasis without inhibiting the tumor suppressor function of CAV1, Y14 phosphorylation emerges as an attractive therapeutic target to prevent metastasis without altering beneficial traits of CAV1.

## INTRODUCTION

Melanoma is one of the most common cancers and its global incidence has increased significantly during the last decades. Because malignant melanomas are highly metastatic and generally resistant to current chemotherapeutic treatments, this type of cancer is associated with high mortality rates in these patients [1].

In order to metastasize, tumor cells must develop specific characteristics that permit detachment of cells from the matrix within the primary tumor, local migration and invasion of stromal tissue, intravasation into blood vessels, survival in the circulatory system and extravasation, local invasion of the secondary site(s), attachment, perhaps dormancy, and finally proliferation and secondary tumor formation [2, 3]. Each one of these events requires specific molecular components in tumor cells, the surrounding extracellular matrix (ECM) and in stromal cells [4]. The underlying essential interactions involve cell-ECM and cell-cell contacts, which are processes stimulated by secreted factors [3]. Fibronectin and laminin are well-characterized, non-collagenous ECM glycoproteins important for cell adhesion. Both have domains with unique functions that promote binding to specific collagens and proteoglycans, as well as to cell surfaces [5–7]. The presence of such proteins enhances migration of B16 melanoma cells in Boyden chamber assays, where filters are precoated with either one of these glycoproteins as an attractant [8]. These experiments indicate that tumor cell migration is favored by haptotaxis towards immobilized attractant proteins, implicating non-collagenous, adhesive glycoproteins located in the interstitial space and on the basement membranes in directly promoting the invasion of some metastatic cell types *in vivo*. The best-characterized receptors for these ECM glycoproteins are the integrins.

Integrins are the most important adhesion proteins in cell-matrix interactions and therefore represent key molecules involved in the stimulation of cell adhesion, invasion and motility processes [9]. Consistent with these observations, changes in their expression are often associated with tumor progression [10]. In human melanomas, beta1 and beta3 integrins increase during metastasis and high levels have been detected in the vertical growth phase of many primary melanomas [11–15]. The relevance of these observations is further underscored by reports highlighting the importance of beta1 integrins in melanoma cell migration and the associated matrix reorganization [14, 16–19]. Thus, specific cell-ECM interactions are strongly implicated in melanoma malignancy.

Endocytosis and recycling of integrins and ECM components are important events in tumor invasion and metastasis [20, 21]. Moreover, remodeling of fibronectin matrix through endocytosis of beta1 integrin involves the protein Caveolin-1 (CAV1) [22, 23], the expression of which correlates with progression of several human cancers [24–26], including melanomas [27–29]. Functional integrins are heterodimers containing an alpha and a beta subunit and the dimer composition determines integrin binding specificity. One of the preferred partner subunits for beta1 integrin is alpha5 and the heterodimer binds preferentially to fibronectin. In the highly metastatic B16F10 melanoma cells, alpha5 expression is elevated compared to poorly metastatic B16F1 cells and neutralization of this integrin with alpha5-specific antibodies significantly reduces the potential of B16F10 cells to generate pulmonary metastasis in mice and inhibits cell adhesion to fibronectin *in vitro* [30]. Thus, in the current study we evaluated whether CAV1 expression stimulates the surface expression of alpha5 and beta1 integrins in the B16F10 melanomas and to what extent these integrins contribute to CAV1-enhanced migration and invasion reported here.

CAV1 (21-24 kDa) is an integral membrane protein involved in several physiological processes, including caveolae biogenesis [31, 32], cholesterol transport [33], endocytosis [34] and cell signaling [35]. In cancer, CAV1 has been suggested to function as a tumor suppressor in early stages of cancer development and later on as a promoter of metastasis [26, 36] and this ambiguity in function is suggested to depend on the cell type and context [28, 37, 38]. Consistent with a function in metastasis, CAV1 reportedly enhances cell migration in a number of cell types, and does so in a manner dependent on tyrosine-14 phosphorylation by Src family kinases [39–41]. Accordingly, CAV1-enhanced migration is impaired by introducing a non-phosphorylatable phenylalanine into the protein at position 14 (Y14F) [42, 43]. In addition, CAV1 is a crucial regulator of focal adhesion (FA) dynamics, because it promotes FAK stabilization in FAs, thereby favoring their turnover and subsequent cell migration [42, 44]. These data identify phosphorylation on Y14 as being important for CAV1 function in migration. However, the importance of this phosphorylation site in metastatic cells for migration on pure ECM surfaces, its function in experimental lung metastasis of melanomas and particularly whether Y14 mutation might interfere with the tumor suppressor function of CAV1 in the same cells, remained to be defined.

A relevant step in metastasis is the extravasation of tumor cells from the circulatory or lymphatic system and invasion of the new tissue, where the secondary tumors are formed. This event is characterized by transendothelial migration (TEM) of tumor cells through the capillary endothelium, which occurs in a manner similar to that observed for lymphocytes [45]. Adhesion molecules, especially integrins and cell surface glycoproteins, like Cell Adhesion Molecules (CAMs), are key players in this process [46, 47]. The integrin beta1 has been described as important for metastasis in murine and human melanoma cells [48]. As mentioned above, CAV1 increases beta1 integrin surface availability, but whether it promotes TEM of tumor cells and hence, metastasis is currently unknown.

In the present study, we used the B16F10 murine melanoma model and determined the role of individual ECM components and Y14 phosphorylation of CAV1 in cell adhesion, spreading and migration. We also assessed the importance of CAV1 Y14 phosphorylation in invasion, TEM and lung metastasis. Our results indicate that the ECM components fibronectin and laminin (but not vitronectin or collagen) stimulate CAV1 Y14 phosphorylation and that CAV1 promotes melanoma migration on these surfaces, as well as matrigel invasion in a Y14-dependent manner. Additionally, we show that CAV1 Y14 phosphorylation is required to enhance beta1 integrin-dependent TEM and *in vivo* lung metastasis. Importantly, however, CAV1 Y14 phosphorylation is not required for CAV1 tumor suppressor activity. Therefore, phosphorylation of Y14 in the CAV1 protein can be therapeutically targeted to selectively diminish metastasis without inhibiting the tumor suppressor function of CAV1.

## RESULTS

### CAV1-enhanced B16F10 cell migration and invasion is blocked by the Y14F, but restored by the Y14E mutation

**Figure 1:**
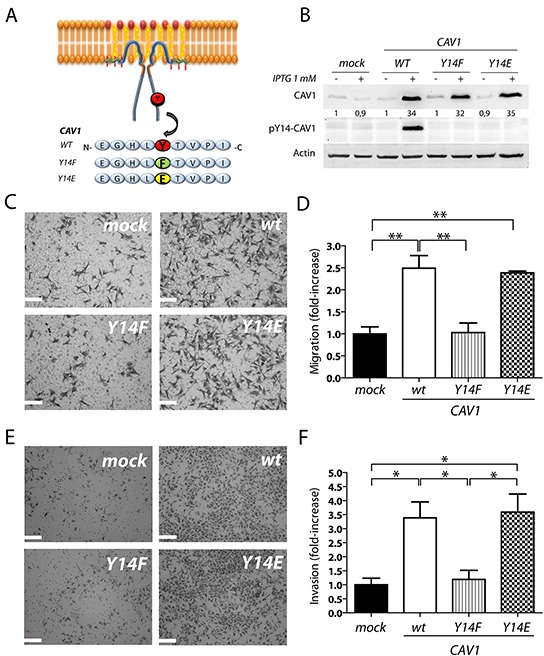
CAV1-enhanced B16F10 cell migration and invasion are dependent on tyrosine 14 **A.** Mutated versions of CAV1 in Y14, (CAV1/Y14F and CAV1/Y14E; non phosphorylatable and phosphomimetic, respectively) generated by site-directed mutagenesis are depicted in a scheme. **B.** B16F10 cells were transfected with empty vector pLacIOP, pLacIOP-(CAV1/wt), pLacIOP-(CAV1/Y14F) or pLacIOP-(CAV1/Y14E) (see Materials and Methods for details) to generate stably transfected B16F10(mock), B16F10(CAV1/wt), B16F10(CAV1/Y14F) and B16F10(CAV1/Y14E) cells, respectively. Post-selection with hygromycin B, cells were induced with 1 mM IPTG for 48 h and treated with 5 mM H_2_O_2_ for 20 min to induce CAV1-phosphorylation on Y14, for analysis by Western blotting. Relative CAV1 levels normalized to β-Actin by scanning densitometry are shown as the fold-increase with respect to the (mock) condition. **C.** and **D.** B16F10 cells (5×10^4^) were added to transwell inserts pre-coated on the lower side with fibronectin (2 μg/ml). Cells were allowed to migrate for 2 h and then detected after fixation on the lower side of the membrane by crystal violet staining. (C) Images of the transwell inserts viewed at 400X magnification are shown (scale bar 100 μm). (D) Data averaged from 6 different fields in three independent experiments and normalized to values for mock cells are shown (mean ± S.E.M, **p<0.01). **E.** and **F.** B16F10(mock), (CAV1/wt), (CAV1/Y14F) and (CAV1/Y14E) cells (5×10^4^) were added to matrigel inserts, allowed to invade for 22 h and then detected and quantified in the same manner as in D. (E) Images of the matrigel inserts viewed at 200X magnification are shown (scale bar 200 μm). (F) Data averaged from 6 different fields in three independent experiments were normalized to values obtained for B16F10(mock) cells (mean ± S.E.M, *p<0.05).

CAV1-phosphorylation on tyrosine 14 by Src family kinases is required to promote migration of fibroblasts [43]. Previous data obtained in B16F10 melanoma and MDA-MB-231 breast cell lines using PP2, a selective pharmacological inhibitor of the Src family kinases, prevented CAV1-enhanced wound closure. Moreover, both endogenous (MDA-MB-231) and ectopically expressed (B16F10) CAV1 failed to undergo phosphorylation in the presence of PP2 [42]. Here, we also observed in a multiple scratch assay that Y14-phosphorylation of CAV1 increased significantly 30 minutes after injuring the monolayer (Supplementary Figure S1). To determine the function of phosphorylated Y14-CAV1 in migration of B16F10 cells, we generated a non-phosphorylatable (Y14F) and a phosphomimetic (Y14E) CAV1 construct by site-directed mutagenesis (Figure 1A). B16F10 cells were then transfected with a plasmid (placIOP) encoding these mutated CAV1 proteins. Upon induction with IPTG, stably transfected cells were found to express equivalent levels of the CAV1(Y14F) and CAV1(Y14E) mutant proteins, as well as wild type CAV1 (Figure 1B). Therefore, subsequently observed differences could not be attributed to differential expression of wild-type CAV1 or the mutated proteins. Treatment of B16F10 cells with hydrogen peroxide, an activator of Src kinases [49] and an important inhibitor of protein tyrosine phosphatases [50], led to CAV1-phosphorylation on Y14 (as revealed by a specific monoclonal antibody), but phosphorylation of CAV1 in mock cells and cells expressing the mutated protein were essentially not detectable (Figure 1B).

As we have previously described [42], expression of CAV1 increased migration of B16F10 cells in Boyden Chamber assays; instead, the non-phosphorylatable CAV1(Y14F) variant failed to do so (Figure 1C and 1D). Importantly, expression of the phosphomimetic CAV1(Y14E) variant enhanced migration to a similar extent as did wild-type CAV1 (Figure 1C and 1D). These results confirm the relevance of CAV1 tyrosine-14 phosphorylation in promoting the migration of B16F10 cells *in vitro*.

Enhanced cell invasiveness is one of the hallmarks of advanced cancers and represents an important step in the sequence of events leading to metastasis [3, 51–53]. To assess the relevance of CAV1 in promoting the invasive phenotype of B16F10 melanomas, we evaluated cell behavior in a Matrigel assay. CAV1 presence in B16F10 cells increased invasion (3,5-fold) while this was not the case for the non-phosphorylatable CAV1(Y14F) mutant (Figure 1E and 1F). CAV1-enhanced invasion was also observed in B16F10 cells expressing the phosphomimetic CAV1(Y14E) variant (Figure 1E and 1F). These results indicate that CAV1-enhanced invasion of B16F10 cells also required Y14 phosphorylation.

### CAV1-increased persistency and directionality of migration on fibronectin and laminin requires tyrosine 14 in B16F10 cells

**Figure 2:**
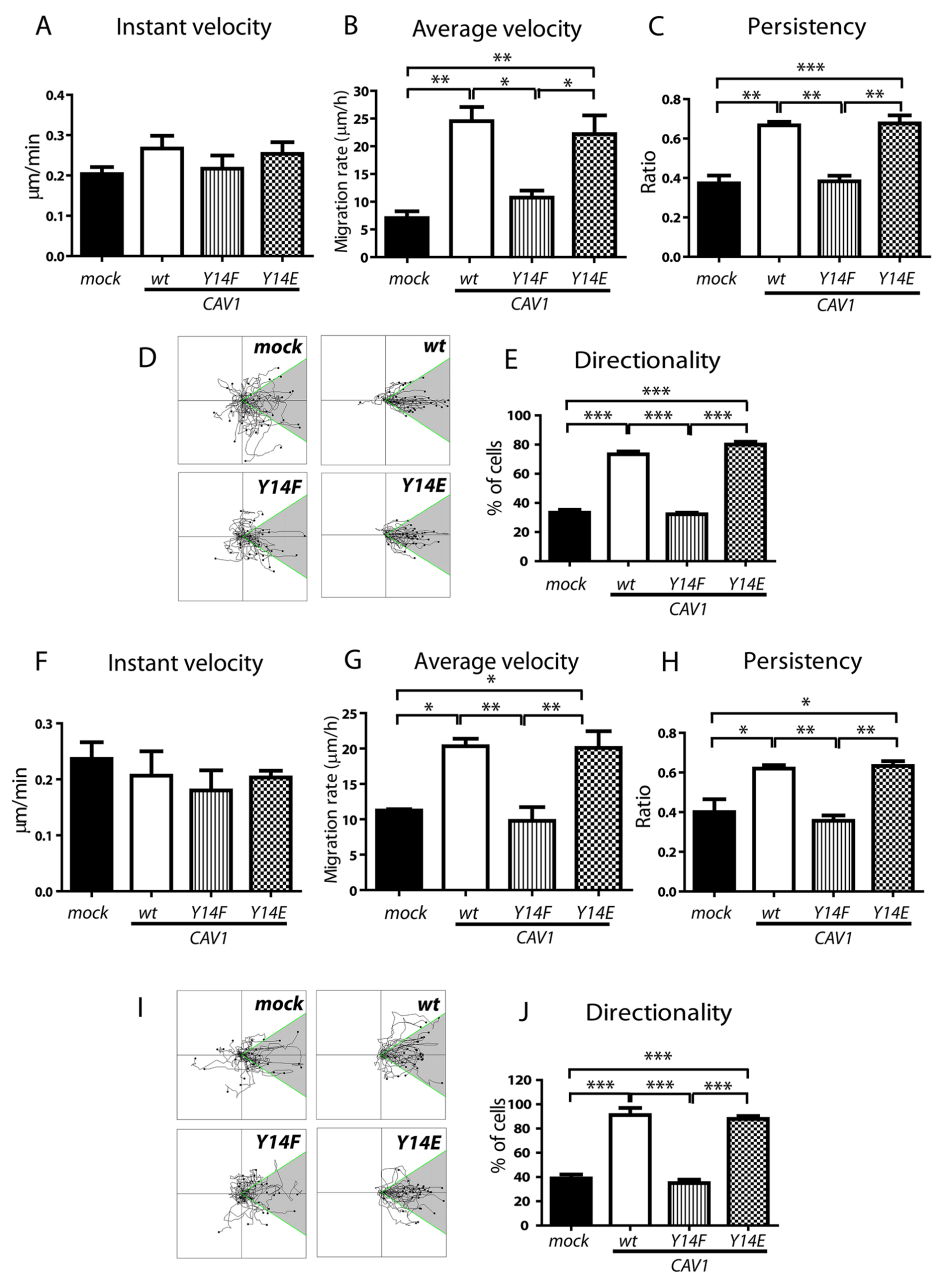
CAV1-increased migration on fibronectin and laminin requires tyrosine 14 in B16F10 cells B16F10(mock), (CAV1/wt), (CAV1/Y14F) and (CAV1/Y14E) cells were induced with IPTG (1 mM) for 48 h. Then, 1×10^6^ cells were seeded in migration micro-devices, pre-coated in the side-channels with fibronectin (50 μg/ml) or laminin (50 μg/ml). Cells were allowed to attach for 2 h in the central chamber. Then, side-channels were filled with culture media and migration was recorded by time-lapse video microscopy for 7 h at 15-min time intervals. Cell tracks were determined using the Image J Software (“Manual Tracking” plug-in). **A.** The Instant velocity (μm/min) at any given time point was analyzed for individual cells during tracking on fibronectin. **B.** The Average velocity was obtained as the quotient between the Euclidean distance (μm) and the total time of migration while tracking the cells on fibronectin. **C.** Persistency of migration was calculated as the ratio between the net distance and the total distance of migration on fibronectin. **D.** Individual cell tracks on fibronectin are shown in a Cartesian coordinate system for each cell type. **E.** Directionality of migration (% of cells) on fibronectin was obtained from D, whereby tracks within a 60° angle with respect to the direction of cell movement were considered as oriented (shaded region). Migration on laminin; **F.** Instant velocity, **G.** Average velocity, and **H.** Persistency of migration on laminin are shown. **I.** Individual cell tracks and **J.** Directionality of migration on laminin were obtained from I, as described above. Graphs show values of each parameter averaged from three independent experiments (mean ± S.E.M, n = 3, ***p<0.001; **p<0.01 and *p<0.05).

Migration of cancer cells through basement membranes and extracellular matrices that contain non-collagenous fibronectin and laminin is an essential step during tumor invasion and metastasis [8, 54, 55]. Our results show that fibronectin is an important haptotactic stimulus for B16F10 cell transmigration in Boyden Chambers [42] (Supplementary Figure S2). Moreover, CAV1 promotes B16F10 cell migration in a wound closure assay by increasing cell motility parameters and this ability depends on the phosphorylation of tyrosine-14 [42]. In the reported experiments, cells migrated on an undefined matrix largely produced by the cells themselves. To study the function of cell-matrix interactions in an unbiased manner, it is critical that the surface is neither contaminated with cell debris nor physically damaged [56]. Thus, we now evaluated migration on virgin surfaces coated with specific ECM molecules using Multichannel Migration Devices (MMDs) combined with individual cell tracking analysis by time-lapse video microscopy (see [56] for methodological details). In these experiments, we observed the behavior of B16F10 cells on surfaces coated with fibronectin, laminin, collagen IV or vitronectin. Expression of CAV1 did not significantly increase Instant velocity on fibronectin (Figure 2A). However, expression of the protein did increase the Average velocity (μm/h) (Figure 2B), Persistency (ratio between the net and total distance, Figure 2C) and Directionality of migration (percentage of cells that move within a 60° angle from the starting point, Figures 2D, 2E). Expression of the CAV1(Y14F) mutant failed to enhance Average velocity, Persistency and Directionality of migration, while all three parameters were promoted by the expression of the CAV1(Y14E) protein (Figures 2B-2E). Similar CAV1-mediated effects were obtained for migration on laminin. CAV1 did not significantly increase Instant velocity (Figure 2F) during cell migration, but did increase the Average velocity (Figure 2G), Persistency (Figure 2H) and Directionality (Figures 2I, 2J) of migration in a tyrosine-14 dependent manner. On the other hand, expression of CAV1 did not increase any of the migration parameters of B16F10 cells either on collagen IV or on vitronectin (Supplementary Figure S3). In summary, these experiments provide compelling evidence that CAV1 increases important migration-associated parameters of metastatic melanoma cells in an ECM-dependent fashion.

### CAV1- phosphorylation on tyrosine 14 during cell adhesion on fibronectin and laminin

**Figure 3:**
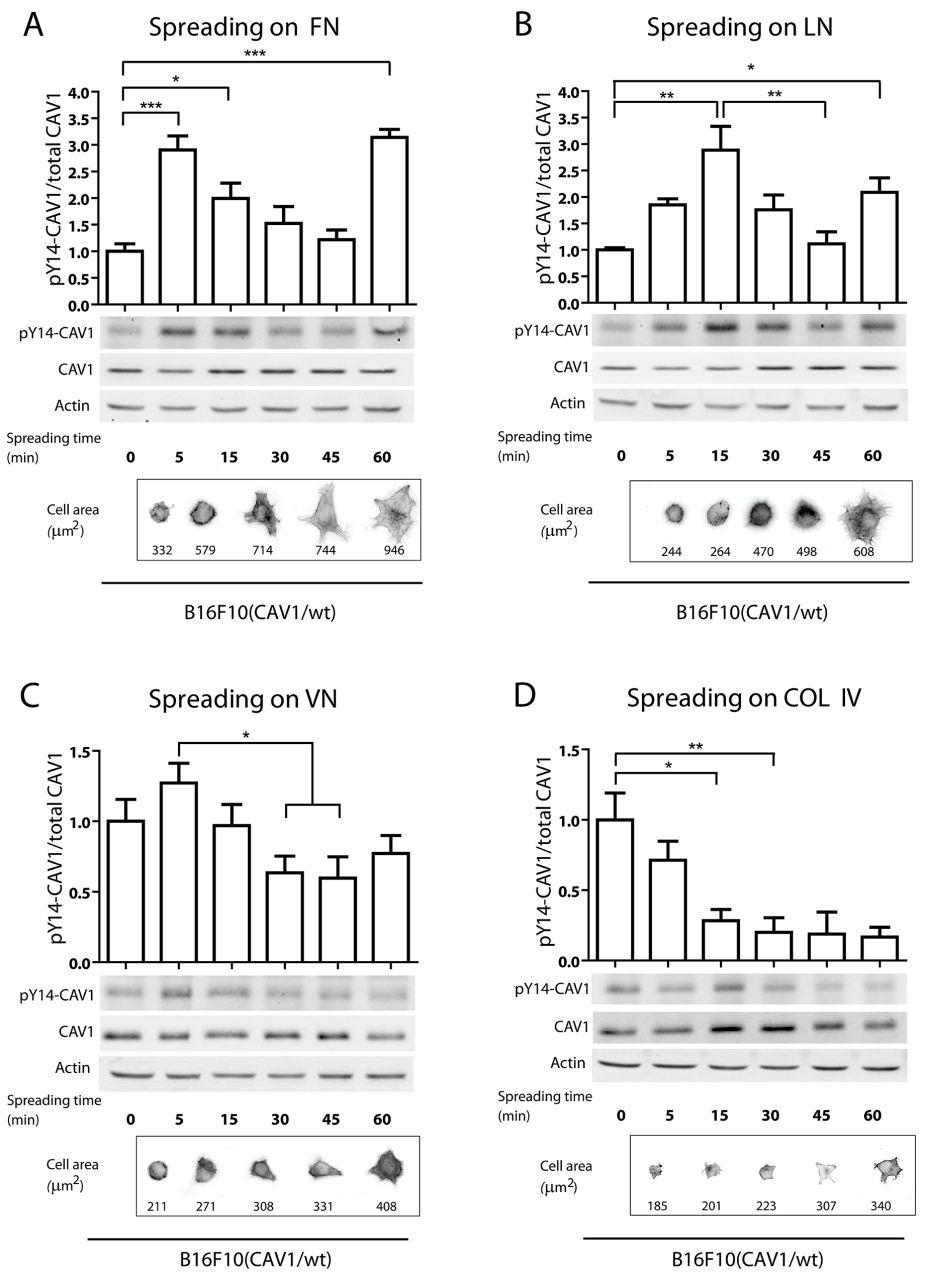
CAV1-phosphorylation on tyrosine 14 during cell spreading on pure ECM surfaces In spreading assays, B16F10(CAV1/wt) cells (1,5×10^6^) were allowed to attach to **A.** fibronectin, **B.** laminin, **C.** vitronectin and **D.** collagen IV-coated plates (2 μg/ml) for different periods of time (0, 5, 15, 30 and 60 min), with time 0 representing cells in suspension. Then, whole cell lysates were prepared and pY14-CAV1 levels were determined by Western blotting. *Upper graphs* show the densitometric analysis of relative pY14-CAV1 levels during cell spreading. *Lower images* show pY14-CAV1, CAV1 and Actin (control) expression by Western Blotting. *Lower panels* show cells in phase contrast and stained with phalloidin during spreading. The average area per cell is indicated in μm^2^. Data shown are the averages from three independent experiments (mean ± S.E.M, n=3,***p<0.001; **p<0.01 and *p<0.05).

Cell-substrate interactions and integrin ligation with the ECM are two events that subsequently trigger focal complex and focal adhesion (FA) formation [57, 58]. Integrin ligation is known to stimulate a number of signaling pathways important for migration that may be linked to CAV1 function. For instance, integrins activate Src, which phosphorylates CAV1 on Y14 [59, 60]. However, it remains yet to be determined how specific ECM-integrin interactions during migration might impact on CAV1-phosphorylation. To assess this, B16F10 cells were seeded on plates covered with pure surfaces of the ECM proteins fibronectin, laminin, collagen IV or vitronectin, and pY14-CAV1 levels were evaluated by Western blotting after different time intervals. Although the presence of CAV1 did not modify overall adhesion of B16F10 cells to these substrates (Supplementary Figure S4), rapid initial phosphorylation of CAV1 on fibronectin (Figure 3A) and laminin (Figure 3B) was detected after 5 and 15 min, respectively. However, no significant increase in Y14-CAV phosphorylation was observed following cell adhesion to vitronectin (Figure 3C) or collagen IV (Figure 3D); instead, basal phosphorylation levels decreased upon adhesion to these substrates. Also, spreading on these two surfaces was substantially reduced (see cell images and surface area for each timepoint) in comparison to the notable increase in surface area observed for cells on fibronectin and laminin. Importantly, a second increase in CAV1-phosphorylation was observed 60 min after adhesion to fibronectin (Figure 3A) and laminin (Figure 3B), but not for the other two substrates (Figure 3C and 3D). These results are consistent with the idea that CAV1 is rapidly phosphorylated upon adhesion of B16F10 cells to preferred ECM substrates, in our experiments fibronectin and laminin, and then again later on when cells initiate migration on those substrates. This interpretation is in agreement with our results identifying CAV1 tyrosine-14 as important in enhancing average velocity, persistent and directional cell migration on pure fibronectin and laminin surfaces (Figure 2).

### Distribution of CAV1 during cell spreading in B16F10 cells

**Figure 4:**
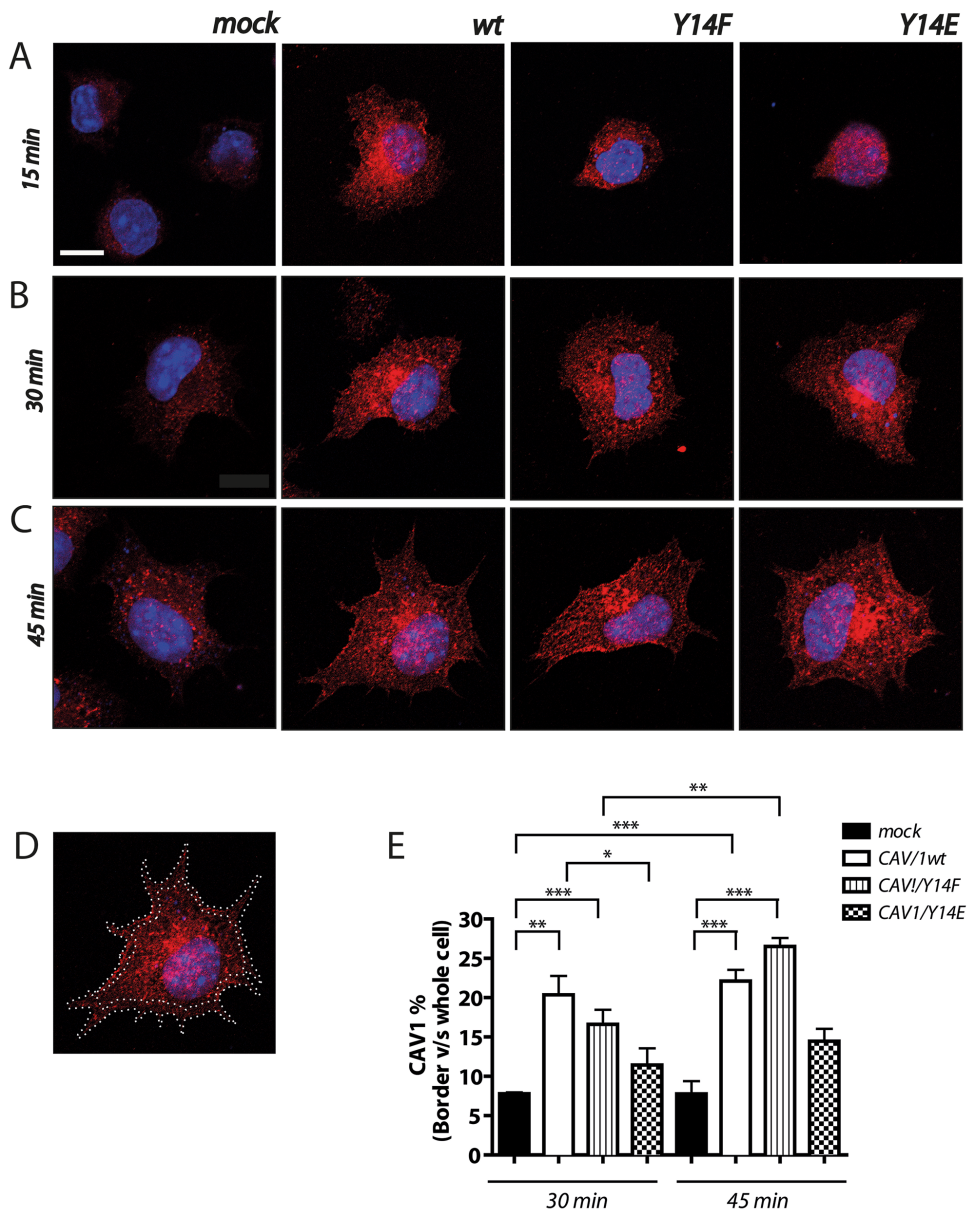
CAV1 distribution during spreading in B16F10 cells B16F10(mock), (CAV1/wt), (CAV1/Y14F) and (CAV1/Y14E) cells were induced with 1mM IPTG for 48 h. Cells were seeded on fibronectin-coated chambered slides (2 μg/ml) and grown in the presence of IPTG (1 mM) for 24 h. Thereafter, cells were serum-starved for 60 min, pulsed with 3% serum and fixed at 15, 30 and 45 min of spreading for CAV1 detection in immunofluorescense experiments. Samples were analyzed with the Fiji Software. **A–C.** Images of B16F10(mock), (CAV1/wt), (CAV1/Y14F) and (CAV1/Y14E) are shown at 15 (A), 30 (B) and 45 (C) min of spreading. Red stain corresponds to CAV1 expression. **D.** Image of B16F10(CAV1/wt) showing ROI definition in the cell periphery. Total fluorescence and ROI (border fluorescence) were quantified at 30 and 45 min of spreading. **E.** In the graph the distribution of CAV1 in the cell periphery is shown in percent (%), calculated as (border fluorescence*100)/whole cell fluorescence). For each experimental condition, at least 5 individual cells were analyzed. Data shown are the averages from three independent experiments (mean ± S.E.M, n=3,***p<0.001; **p<0.01 and *p<0.05).

**Figure 5:**
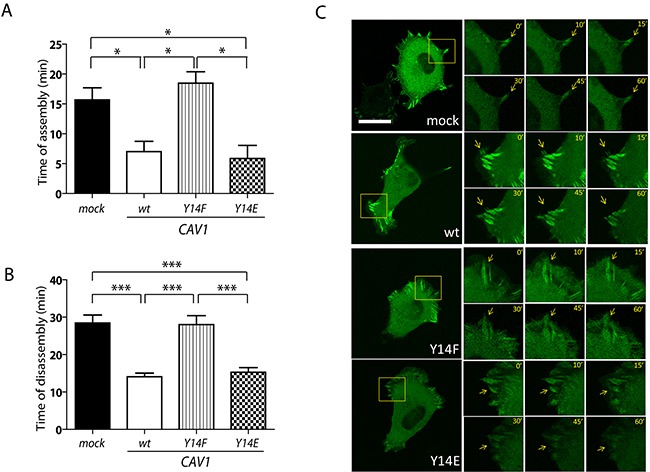
CAV1-enhanced FA dynamics is dependent on tyrosine 14 B16F10(mock), (CAV1/wt), (CAV1/Y14F) and (CAV1/Y14E) cells were induced with 1mM IPTG for 48 h. Cells were transfected with pEGFP-vinculin 24 h prior to the experiment, then seeded on fibronectin-coated chambered slides (2 μg/ml) and grown in the presence of IPTG (1 mM) for 24 h. Thereafter, cells were serum-starved for 60 min, pulsed with 3% serum and recorded by time-lapse video microscopy for 60 min (2-min time intervals). **A.** FA assembly after cells begin to attach to the substrate (time of FA formation); **B.** and **C.** FA disassembly (time of FA disappearance) were measured for at least 10 structures per experiment (scale bar, 50 μm). Note that the kinetics reported in (A) and (B) were obtained from the same set of time-lapse video microscopy experiments. Digital zoom areas in C are shown at selected time points for each cell type. Focal adhesions (FAs, arrows) were defined by size. Images are representative of four independent experiments. Statistically significant differences are indicated (mean ± S.E.M; ***p<0.001 and *p<0.05).

Several reports using fibroblasts, astrocytes and neurons have shown that CAV1 redistributes to the cell rear during migration on substrates in 2D [42, 61–63]. Conversely, in previous studies we observed during 2D migration of metastatic breast cancer (MDA-MB-231) and melanoma (B16F10) cells that polarization of CAV1 was not required, although presence of the protein clearly favors migration [42]. Moreover, migration enhanced by CAV1 is not related to the presence of caveolae in the plasma membrane of B16F10 cells, because these readily detectable structures in fibroblasts, were not detectable in the plasma membrane of CAV1-expressing B16F10 cells, as determined by electron microscopy (Supplementary Figure S5A, S5B y S5C). This result was to be anticipated in the absence of Cavin-1 expression (Supplementary Figure S5D) an essential protein for caveolae biogenesis [64, 65]. Thus, how CAV1 distribution or changes therein relate to CAV1-enhanced migration remain unclear, but appear to strongly depend on the cell under study and the type of migration process that is being analyzed. Because CAV1 promotes FA turnover ([42] and Figure 5) we evaluated changes in CAV1 presence in proximity of the plasma membrane during spreading, an initial step during cell migration. CAV1 distribution was analyzed 15, 30 and 45 min after cell attachment to the substrate. Because after 15 min the still predominantly circular cell morphology did not permit evaluating changes in distribution of CAV1 (Figure 4A) these were quantified only at the later time points 30 and 45 min. Significantly enhanced CAV1 accumulation after these time periods was observed in plasma membrane proximity in the cell periphery for B16F10(CAV1/wt), B16F10(CAV1/Y14F) and B16F10(CAV1/Y14E) (Figure 4B, 4C) as compared to the control condition (mock). In particular for CAV1(wt) and CAV1(Y14F) peripherally accumulating CAV1 lead to clearly discernible plasma membrane labeling while this was less evident for the CAV1(Y14E) mutant. To assess CAV1 translocation from more central cell regions to the periphery, pixel accumulation within a narrow sub-membrane zone (Figure 4D) was quantified, as described (see Materials and Methods). After 30 min peripheral CAV1(wt) accumulation reached a plateau while for the CAV1(Y14F) mutant increases were still detectable until 45 min, although the extent of accumulation for these two proteins was quite similar. Alternately, the CAV1(Y14E) mutant accumulated at a notably slower rate in the cell periphery than the other two CAV1 proteins (Figure 4E).

### CAV1 promotes focal adhesion dynamics in a manner dependent on tyrosine 14 in B16F10 cells

CAV1 has been suggested to participate in FA turnover [42–44, 66]. Thus, we evaluated here the effect of CAV1 Y14 mutations on FA dynamics in B16F10 cells, by transfection with EGFP-vinculin followed by time-lapse video microscopy analysis, as previously reported by our group [42]. CAV1 mutations did not appear to significantly alter the steady-state distribution of the protein in the FA-enriched fraction, although the CAV1(Y14E) mutant did seem to accumulate there to a slightly greater extent (Supplementary Figure S6), likely due to interaction with elements of the cytoskeleton, other than FAs, present in such preparations (see discussion). CAV1 and CAV1(Y14E) expression accelerated the appearance of FAs in B16F10 cells, as compared to CAV1(mock) and CAV1(Y14F) cells (Figure 5A). Moreover, a significant increase in the kinetics of FA disassembly was observed in CAV1 and CAV1(Y14E) expressing cells when compared to the mock control and CAV1(Y14F) cells (Figure 5B; Figure 5C, arrows).

We also determined whether CAV1 was detectable in FAs by co-distribution analysis in immunofluoresence experiments using anti-CAV1 and anti-vinculin antibodies (Supplementary Figure S7). Analysis of the images revealed substantial co-distribution of overexpressed CAV1/wt and CAV1/Y14F with vinculin in B16F10 cells after 30 and 45 min of spreading as compared to B16F10 (mock) cells. Interestingly, for B16F10(CAV1/Y14E) transfected cells, codistribution with vinculin was slower and appeared somewhat less pronounced (Supplementary Figure S7A and S7B; see distribution profiles). Note that no significant changes in either FA size or FA number per cell were observed in B16F10 cells expressing CAV1 constructs (Supplementary Figure S7C and S7D). Taken altogether, these data support the notion that CAV1 localizes to FAs and favors both FA formation and turnover in B16F10 cells in a Y14-dependent manner, without affecting FA area and number.

### CAV1 Y14 promotes wound closure and transendothelial migration through beta1 integrin mediated interactions

**Figure 6:**
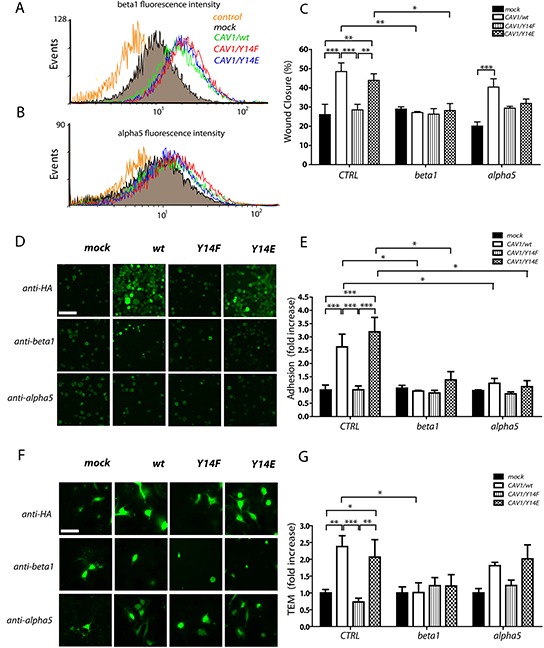
CAV1-enhanced wound closure and transendothelial migration require tyrosine 14 and beta1 surface expression in B16F10 cells B16F10(mock), (CAV1/wt), (CAV1/Y14F) and (CAV1/Y14E) cells were induced with 1mM IPTG for 48 h. Cells were then trypsinized, fixed and immunostained for beta1 and alpha5 integrins and analyzed by flow cytometry. **A.** Beta1 integrin fluorescence intensity. **B.** alpha5 integrin fluorescence intensity. **C.** Confluent monolayers of B16F10 cell lines were wounded with a pipette tip, incubated with anti-beta1 or anti-alpha5 integrin antibodies (5 μg) and images were recorded at 0 and 7 h post-wounding. As a control (CTRL), a non-related anti-GFP antibody was used. The wounded area was measured with the Adobe Photoshop software and the percentage (%) of wound closure in 7 h is plotted for the indicated condition. **D.** EA.hy926 cells (2,5 × 10^5^) were seeded on 24-well plates and impermeable cell monolayers were allowed to form for 72 h. B16F10(mock), (CAV1/wt), (CAV1/Y14F) and (CAV1/Y14E) cells (5×10^4^), previously stained with CellTracker green and incubated for 1 h with anti-HA (CTRL), anti-beta1 or anti-alpha5 integrin antibodies, were added to the EA.hy926 monolayer. Then, B16F10 cells were allowed to adhere to the EA.hy926 monolayer for 1 h (scale bar, 100 μm). **E.** The graph represents the average for adhesion (cells per field) following incubation of the B16F10 cells with the antibodies mentioned above. **F.** EA.hy926 cells (2,5 × 10^5^) were seeded on the Transwell inserts and impermeable cell monolayers were allowed to form for 72 h. B16F10 cell lines (5×10^4^), previously stained with CellTracker green and incubated for 1 h with the neutralizing antibodies using the same procedure described above, were added to the EAhy monolayer in the inserts. Then, B16F10 cells were allowed to penetrate the EA.hy926 monolayer for 6 h. B16F10 cells observed by epifluorescence microscopy with a 40X objective on the lower side of the Transwell membrane are shown (scale bar, 50 μm). **G.** Values in the graph represent the average of TEM (cells per field) following incubation of the in B16F10 cells with the different antibodies. Data were normalized to values obtained for control (mock) cells. Adhesion and TEM was quantified as cells per field from 10 different fields in three independent experiments (mean ± S.E.M, n=3, ***p<0.001; **p<0.01 and *p<0.05).

Integrins are the main cellular receptors for ECM proteins and are important components of FA [58, 67]. In addition, CAV1 is important for integrin-dependent fibronectin adhesion and focal adhesion kinase (FAK) activation [68]. Also, CAV1 promotes alpha5beta1 integrin/fibronectin endocytosis and ECM turnover during extracellular remodeling [22]. Thus, CAV1 appears to be a general regulator of integrin function. Moreover, interaction between alpha5 integrin and fibronectin promotes melanoma metastasis in the B16F10 model [30, 69]. With this in mind, we next evaluated beta1 and alpha5 integrin surface expression and their function in migration of B16F10 melanoma cells. The presence of CAV1 did not increase the total amount of these integrins compared to control (mock) cells, as tested by Western blotting (Supplementary Figure S8), but did increase beta1 and alpha5 integrin surface expression in B16F10 cells as detected by flow cytometry analysis of non-permeabilized cells (Figure 6A and 6B, respectively). These effects on specific integrins were independent of Y14 because the surface expression of beta1 and alpha5 integrins increased both in the presence of wild-type CAV1 and Y14 mutated versions of the protein. It should be noted that no CAV1-dependent changes in beta3 integrin surface expression were observed (Supplementary Figure S8).

To determine the relevance of such elevated beta1 and alpha5 integrin surface expression in migration promoted by CAV1 in B16F10 cells, we employed neutralizing anti-beta1 and anti-alpha5 integrin antibodies while evaluating migration in the wound-healing assay. Wound closure was analyzed 7 h after the addition of antibodies. In control (CRTL) experiments, cells were incubated with a non-related anti-GFP antibody. The presence of CAV1 in B16F10 cells increased wound closure from 25% to 50% in comparison to mock cells, indicating that CAV1 promoted cell migration (Figure 6C). Expression of the mutated CAV1(Y14F) protein prevented this effect, while migration was enhanced by expression of CAV1(Y14E) to a similar extent as observed for the wild type CAV1 protein. Antibody-mediated blocking of beta1 integrin function prevented wild-type CAV1 and CAV1(Y14E)-enhanced B16F10 migration, but the same antibody had no effect on the basal migration of B16F10 cells (Figure 6C). Addition of the anti-alpha5 integrin antibody reduced migration slightly, as compared to control (CTRL) conditions, for mock and wild-type CAV1 cells. However, the difference between these two remained significant, indicating that CAV1 promoted migration even in the presence of anti-alpha5 integrin antibody. For CAV1(Y14F)-expressing cells, migration following incubation with this antibody was slightly elevated as, compared to the migration of mock cells and similar to that observed for CAV(Y14E) cells (Figure 6C). Taken together, these results indicate that the beta1 integrin is important for cell migration promoted by CAV1 in wound healing assays; alternatively, alpha5 integrin does not appear relevant to migration of B16F10 cells in this context. It should be noted that in migration experiments with the anti-beta3 antibody, cells detached after 7 hours and migration could not be analyzed (data not shown).

Transendothelial migration (TEM) is the process by which tumor cells extravasate from the vascular system and invade a specific tissue. Alpha4beta1 integrin is usually expressed in lymphocytes and, as a first step of extravasation, binds to VCAM-1 expressed on activated endothelial cells [45]. Expression of beta1 integrin on melanomas may therefore allow the tumor cells to mimic lymphocytes and facilitate TEM. Here, we investigated the function of CAV1 and increased beta1 and alpha5 expression in adhesion to endothelial cells and TEM of B16F10 cells. TEM experiments were assayed on EA.hy926 cells (an immortalized hybrid of HUVEC and the A549 human lung carcinoma line). EA.hy926 is one of the most commonly used and best-characterized endothelial cell lines that exhibit many endothelium-specific properties and form capillary-like structures in Matrigel [70, 71]. This immortalized cell line was employed to avoid problems associated with the use of primary endothelial cells in culture. In initial experiments, we first established a monolayer of EA.hy926 endothelial cells and tested for monolayer permeability at different time points (24, 48 and 72 h) using a high molecular weight dye that cannot permeate the cell monolayer once tight junctions are formed between all cells. Indeed, after 48-72 h in culture, the dye was no longer able to permeate the cell monolayer (Supplementary Figure S9), indicating the presence of a sealed cell monolayer. To evaluate the effect of CAV1 on B16F10 adhesion to the endothelial monolayer, cells were labeled with a fluorescent dye and seeded onto EA.hy926 cells. Adhesion was quantified as cells per field after 1 h (Figure 6D). Interestingly, CAV1 promoted adhesion to the endothelial monolayer in a Y14-dependent manner (Figure 6D and 6E), although *in vitro* the presence of CAV1 did not modify the adhesion of B16F10 cells to pure ECM surfaces (Supplementary Figure S4). To assess the relevance of integrins in B16F10 adhesion to the endothelial monolayer, the cells were incubated with the blocking anti-beta1 and alpha5 integrin antibodies and adhesion was analyzed in the same manner as described above. In control (CRTL) conditions, cells were incubated with a non-related anti-HA antibody. These experiments revealed that both blocking antibodies reduced CAV1 and CAV1(Y14E)-enhanced adhesion to EA.hy926 cells (Figure 6D and 6E) compared to the control condition.

To evaluate TEM, B16F10 cells labeled with a fluorescent dye were seeded onto a monolayer of EAhy926 cells grown for 72 h and allowed to transmigrate across the monolayer for 6 h. The ability of B16F10 cells to transmigrate through an EA.hy926 monolayer was significantly increased in the presence of CAV1 and CAV1(Y14E) (Figure 6F and 6G). Interestingly, this effect was prevented by expression of CAV1(Y14F) in cells. These data indicate that CAV1 enhanced TEM in a Y14-dependent manner. In this same assay, we then evaluated, using blocking antibodies, the contributions of beta1 and alpha5 integrin surface expression to TEM of B16F10 cells. As suspected based on previous data, incubation of wild-type CAV1 and CAV1(Y14E) expressing cells with an anti-beta1-integrin antibody significantly reduced the ability of CAV1 to enhance TEM as compared to the control condition (Figure 6F and 6G). Addition of the anti-alpha5 integrin antibody did not induce significant changes in TEM of B16F10 cells compared to the control (Figure 6F and 6G). These results suggest an important function for phospho-CAV1 in regulating metastatic extravasation, whereby particularly surface expressed beta1 integrins were identified as being essential for adhesion to vascular endothelium and TEM. Also, a function for alpha5 integrin in endothelium adhesion is unveiled here; however, alpha5 appeared not to be crucial in melanomas for 2D or 3D migration.

### CAV1-enhanced metastasis of B16F10 cells requires phosphorylation on tyrosine 14

**Figure 7:**
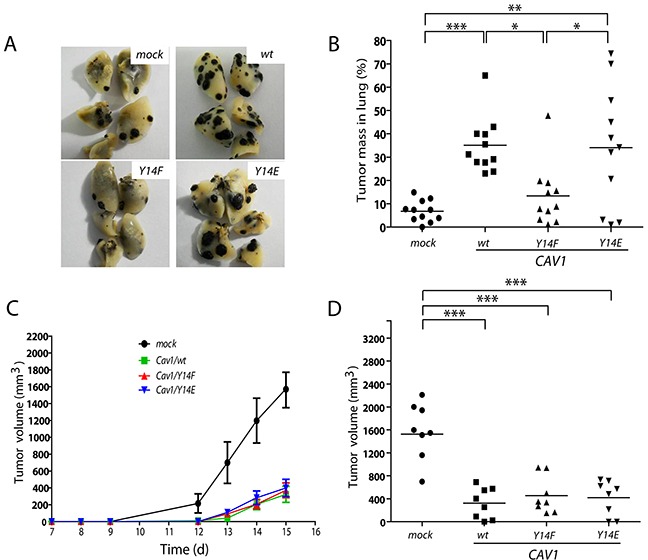
CAV1-enhanced lung metastasis of B16F10 melanoma cells is dependent on tyrosine 14 C57BL/6 mice were intravenously injected with B16F10(mock), (CAV1/wt), (CAV1/Y14F) or (CAV1/Y14E) cells (5×10^5^), previously grown for 48 h in the presence of IPTG (1 mM). **A.** The images show the black metastatic lung mass after sacrificing the animals at day 21. **B.** The graph shows the results of 44 mice in total (11 per group). The lung tumor mass in C57BL/6 mice for B16F10(mock), B16F10(CAV1/wt), B16F10(CAV1/Y14F) and B16F10(CAV1/Y14E) cells was 7%, 35%, 13% and 34%, respectively. **C.** and **D.** C57BL/6 mice were subcutaneously injected with B16F10(mock), (CAV1/wt), (CAV1/Y14F) or (CAV1/Y14E) cells (1×10^5^) previously grown for 48 h, in the presence of IPTG (1 mM). (C) Tumor volume (mm^3^) was monitored in each animal. Results shown are the average from data obtained with 8 mice per group between day 7 and day 15. (D) The average tumor volumes (mm^3^) measured on day 15 for the 4 groups of animals (32 in total) are shown. For CAV1/wt, CAV1/Y14F, CAV1/Y14E expressing cells and mock-transfected controls, tumor volumes were 1693 mm^3^ (S.D ± 655); 323 mm^3^ (S.D ± 252); 455 mm^3^ (S.D ± 298) and 418 mm^3^ (S.D ± 284), respectively. Statistically significant differences are indicated (***p<0.001; **p<0.01 and *p<0.05).

Increased CAV1 expression has been correlated with metastasis in a number of human cancers [24–26]. The function of CAV1 in metastasis is associated with its ability to promote cell migration, which requires phosphorylation on tyrosine-14 [42, 72]. We have previously determined the importance of CAV1 in promoting lung metastasis using a B16F10 melanoma model in syngeneic C57BL/6 mice and reported that overexpression of CAV1 in B16F10 cells led to increased lung metastasis compared with control cells [28]. To determine the function of CAV1 phosphorylated on tyrosine 14 in metastasis of B16F10 cells, we injected mice with B16F10 cells transfected with wild-type CAV1 or the mutated versions of CAV1. As expected, expression of wild-type CAV1 significantly increased lung metastasis of B16F10 cells injected intravenously into syngeneic C57BL/6 mice, as compared to B16F10 (mock) cells (Figure 7A and 7B; images *wt* and *mock*, respectively). Interestingly, expression of CAV1(Y14F) in B16F10 did not increase metastasis beyond the levels observed with CAV1(mock) cells, whereas for CAV1(Y14E) expressing B16F10 cells, metastasis was enhanced to the same extent as seen for CAV1 wild-type expressing B16F10 cells (Figure 7B).

To rule out the possibility that mutations of Y14 may have inhibited CAV1 function in a non-specific manner, we determined whether they altered a different feature of CAV1, namely its tumor suppressor function [28]. To this end, we assessed subcutaneous tumor growth of B16F10 cells expressing wild-type CAV1, CAV1(Y14F) or CAV1(Y14E). As we have previously described [28, 29], expression of wild-type CAV1 in B16F10 cells delayed tumor formation as compared to B16F10(mock) cells in C57BL/6 mice (Figure 7C). Importantly, the same was observed with both mutant versions of the protein. Tumors formed by B16F10 cells expressing wild type and mutated CAV1 were significantly smaller at day 15 post-subcutaneous injection, compared with tumors from animals injected with B16F10(mock) cells (Figure 7D). No significant differences were found between tumors of B16F10 cells expressing wild type and mutated versions of CAV1.

In conclusion, the function of CAV1 as a tumor suppressor is not altered by CAV1 mutations in Y14. Importantly, the dual role of CAV1 as tumor suppressor and promoter of metastasis in this experimental model represent completely independent functions of this protein, suggesting it should be possible to target the undesirable function of CAV1 as a metastasis promoter without inhibiting its beneficial trait as a tumor suppressor.

## DISCUSSION

The function of CAV1 in cancer and specifically in cell migration, invasion and metastasis remains a controversial issue. A large body of evidence favors the notion that CAV1 function as a tumor suppressor or promotor of metastasis is cell context dependent. Our group has previously shown that a) CAV1 function as a tumor suppressor is conditioned by the expression of E-cadherin and that presence of the latter blocks CAV1-enhanced lung metastasis [28, 29, 37]; b) the expression of CAV1 in metastatic cells lacking E-cadherin enhances cell polarization, directional migration and cell persistency [42], and c) CAV1 enhanced migration of B16F10 cells in Transwell assays is not observed upon expression of the Y14F mutant protein [42]. In the present study, we further evaluated the importance of the Y14 residue in lung metastasis. Our results show that wild-type CAV1 and CAV1(Y14E), but not CAV1(Y14F) enhanced migration, invasion, TEM *in vitro* and lung metastasis *in vivo*. Furthermore, this ability was linked to surface expression of a beta1 integrin and the interaction with ECM components prevalent in the lung, such as fibronectin and laminin.

Importantly, however, introducing these mutations to the Y14 residue had no effect on the ability of CAV1 to function as a tumor suppressor. Thus, these studies not only confirm the relevance of CAV1 Y-14 in migration- and metastasis-related events, but also show that the two functions of CAV1 as a tumor suppressor and promoter of metastasis can be ascribed to separable intrinsic traits of the protein.

*In vivo*, the expression of CAV1 in B16F10 melanoma cells enhances metastasis to the lungs of C57BL/6 mice [28]. In the present study, we determined the requirement of CAV1-phosphorylation on Y14 to enhance the metastatic phenotype in B16F10 cells. In the respiratory tract, fibronectin and laminin are important ECM glycoproteins that contribute to development and morphogenesis. Fibronectin is a major component of the connective tissue and is located in the respiratory tract around the capillaries and the basement membrane of the alveolar epithelium [73]. On the other hand, laminin is the most important glycoprotein in basal membranes [74]. Moreover, pre-incubation of metastatic murine melanoma cells with syngeneic whole laminin followed by tail vein injection increased tumor cell retention in the lung and strongly stimulated metastasis [75]. Consistent with these observations, we show here that CAV1-enhanced average velocity, directional and persistent migration of B16F10 cells in a Y14-dependent manner, specifically on fibronectin and laminin (Figure 2), but not on other surfaces, such as collagen IV or vitronectin (Supplementary Figure S3). These preferences of CAV1 expressing cells contribute to tumor cell attachment and metastasis formation in the lungs of mice. In this context, it is important to note that cell adhesion per se to pure ECM proteins (i.e. fibronectin) did not depend on CAV1 expression, excluding the possibility that changes in migration observed *in vitro* were associated with differences in cell adhesion (Supplementary Figure S4).

The results obtained analyzing Y14-phosphorylation of CAV1 suggest a function for specific integrins that activate Src in enhancing initial CAV1-phosphorylation (see first peak), upon cell adhesion [59]. The second increase in CAV1 Y14-phosphorylation is likely to mediate enhanced cell migration on fibronectin and laminin, attributable to enhanced FA turnover observed in B16F10 cells expressing CAV1. Consistent with this interpretation, beta1 integrin activation reportedly stimulates CAV1-phosphorylation, as beta1 integrin-blocking antibodies inhibit shear stress-induced CAV1-phosphorylation and actin reorganization in bovine aortic endothelial cells [76]. However, more experiments are required to identify the additional integrin subunits that contribute to CAV1-phosphorylation on Y14 and thereby promote migration on pure ECM surfaces.

Persistent and directional migration depends on dynamic formation and turnover of adhesions mediated by integrins, in addition to polarized assembly and disassembly of these structures [77]. These observations are consistent with our model, where pY14-CAV1 promotes FA assembly and disassembly (Figure 5). Here, it is important to note that around 50% of total CAV1 accumulated in FA-enriched extracts, and mutations on Y14 did not modify substantially this distribution, although the CAV1(Y14E) mutant accumulated there to a slightly greater extent (Supplementary Figure S6). This preparation likely contains many cytoskeletal components beyond those present in FAs and thus, accumulation in this fraction may be attributable to increased interaction of the phosphomimetic version with proteins not necessarily present in FAs. This interpretation is supported by the observation that the CAV1(Y14E) mutant moves to the cell periphery and co-distributes there with the FA marker vinculin at a notably slower rate than wild type CAV1 or CAV1(Y14F) (Supplementary Figure S7). In general, however, our results are in agreement with reported data showing changes in FA dynamics upon CAV1 expression [66, 78]. Also, our results coincide with previous observations indicating that CAV1 co-distributes with FAs. Furthermore, in mammary carcinoma cells, phospho-CAV1, together with the Mgat5/Gal-3 lattice, stabilizes alpha5-integrin, cytosolic FAK and paxillin, in FAs, thereby promoting FA disassembly and turnover, as well as stimulating cellular displacement and motility [66]. Here also, increased cell motility of CAV1 expressing cells is associated with increased FA dynamics. In the same study, the function of phospho-CAV1 was evaluated by the expression of a phosphomimetic Y14D version of the protein. In our study, we replaced tyrosine-14 by glutamic acid (E) instead of aspartic acid (D). Nonetheless, we obtained similar results concerning the function of the phosphomimetic CAV1 in FA turnover (Figure 5), as well as in cell migration (Figure 1C and 1D). In conjunction, these observations point towards the relevance of negative charge at this site, possibly to unfold the NH2-terminal protein structure of CAV1 and thereby facilitate interactions with the CAV1 scaffolding domain [79] rather than to permit direct binding of partner proteins via SH2 domains, as would be predicted for phosphorylation on tyrosine. While intriguing, further experiments are required to sustain this possibility.

CAV1 is highly prevalent in the interior of B16F10 cells (Figure 4) and does not polarize during cell migration [42]. However, in B16F10 cells, migration was associated with enhanced presence of CAV1 in the cell periphery and co-distribution with vinculin, a marker of FAs. These observations are consistent with the data showing that CAV1 modulates FA turnover (Figure 5). Interestingly, the CAV1(Y14E) mutant tended to accumulate at a slower rate in the cell periphery and FAs during spreading/migration. These observations may explain why the CAV1(Y14E) mutant is not more effective in promoting migration of B16F10 cells than the wild type CAV1 protein.

CAV1 increased migration in B16F10 cells lacking Cavin-1 expression and caveolae in the plasma membrane (Supplementary Figure S5). These observations are in agreement with previous findings indicating that the presence of caveolae and Cavin-1 (a regulator of CAV1 function) in metastatic cells inhibits migration enhanced by CAV1 [80, 81]. Thus, the precise nature of the plasma membrane-associated CAV1 pool remains elusive, although some of the protein is present in FAs. Also, additional experiments are required to determine the origin of CAV1 prior to accumulation in proximity of the plasma membrane and to what extent CAV1 is required there to enhance migration in metastatic cells.

Formation, maturation and disassembly of FAs are basic prerequisites of cell migration and are dependent on the recruitment, signaling and endocytosis of integrins [82]. Moreover, an increase in the expression of alphaVbeta3 integrin correlates with cell migration, intravasation and metastasis of melanomas [83–85]. Furthermore, the expression of alpha5beta1 integrin is required for lung colonization by B16F10 melanoma cells in C57BL/6 mice [69] and CAV1 has been suggested to participate in the regulation of integrins by a variety of mechanisms [86]. Particularly, phospho-CAV1 is important in endocytosis of these proteins [87], which contributes to protein turnover at the cell surface. Our analysis by flow cytometry revealed that CAV1 increased surface expression of beta1 (Figure 6A) and alpha5 (Figure 6B) integrin in a Y14-independent manner. However, only beta1 integrin was required for CAV1-enhanced migration, because a neutralizing anti-beta1 antibody prevented migration enhanced by CAV1 (Figure 6C), even in experiments where the phosphomimetic version of the protein was expressed. It should be noted that application of neutralizing anti-beta3 integrin antibody led to complete detachment of cells, demonstrating the importance of this integrin in the adhesion of melanoma cells. These results highlight the relevance of beta1 integrin in CAV1-driven migration. Anti-alpha5 integrin antibodies did decrease adhesion to endothelial cell monolayers, the first step required for TEM; however, the apparent reduction in TEM was not statistically significant. Potentially, beta1 integrin may interact with alpha5 subunits, to favor cell adhesion and migration of B16F10 cells; however, additional binding partners are likely to participate as well.

Data from several publications concerning heterodimeric integrin expression have shown that the levels of alpha3beta1, alpha4beta1, alpha5beta1 and alphaVbeta3 integrins seem to increase in primary and metastatic melanomas. Alternatively, a significant decrease in alpha1beta1, alpha2beta1 and alpha6beta1 integrins has been reported in metastatic melanomas compared to primary melanomas [12, 13, 48, 88]]. Importantly, alpha4beta1 and alpha5beta1 integrins are receptors for fibronectin, while alpha3beta1 binds to laminin [89], the two ECM components shown here to promote specific Y14-phosphorylation of CAV1 and cell spreading/migration in B16F10 cells. Considering these observations and our results, we propose that alpha5 may be one of several beta1 binding partners involved in melanoma adhesion to endothelial cells and TEM. However, further experiments are required to determine if alpha3 and alpha4 integrins are upregulated in B16F10 cells expressing CAV1 and to what extent these proteins may contribute to CAV1-enhanced TEM.

**Figure 8:**
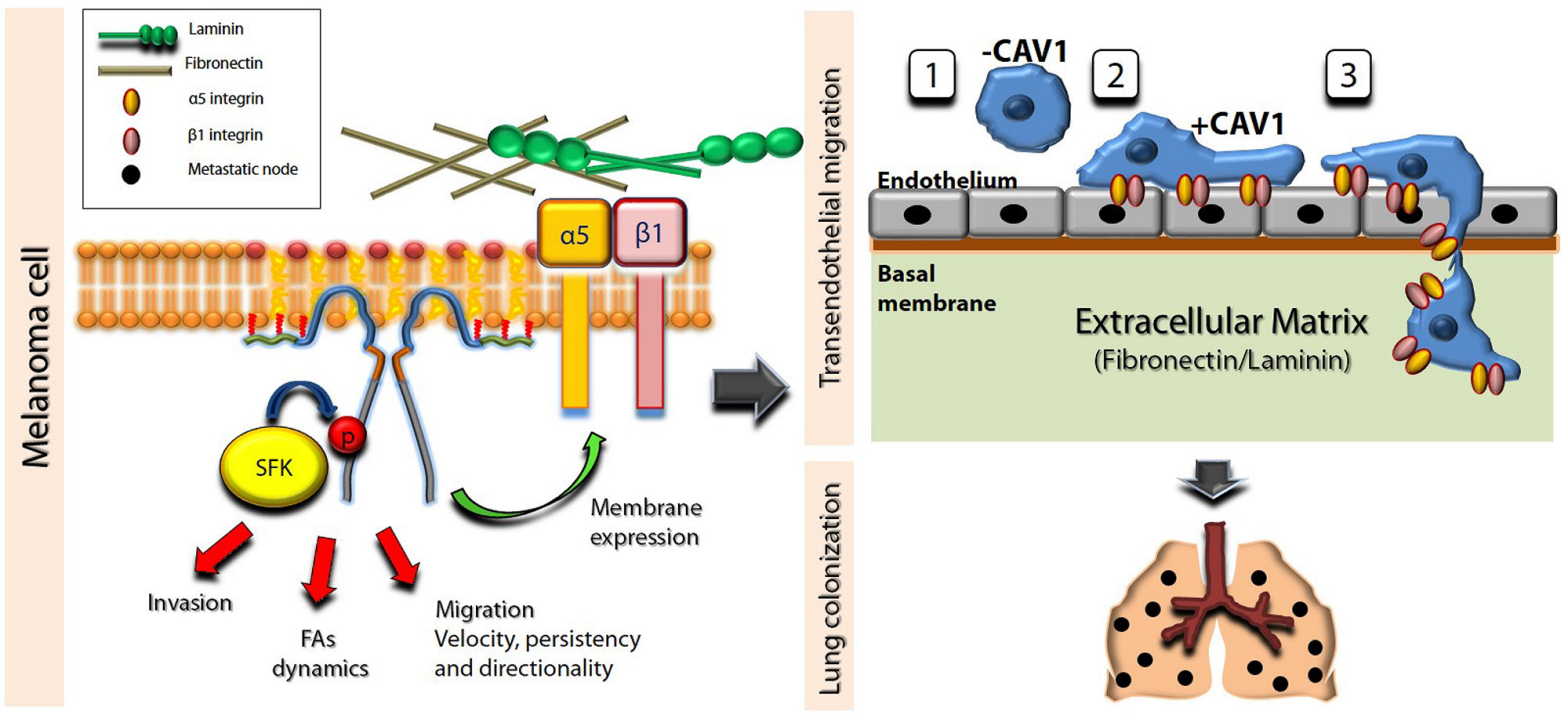
Schematic summary of data showing that CAV1-enhanced migration, invasion, TEM and metastasis are dependent on tyrosine 14 and membrane expression of beta1 (and alpha5) integrins CAV1 is shown as a dimer associated with the plasma membrane. For simplicity, higher states of oligomerization were not considered. Specific ECM-integrin interactions stimulate phosphorylation of CAV1 on Y14 mediated by Src family kinases (SFK), which favors FA dynamics, migration and invasion by melanoma cells. Note that alpha5 is shown here as the beta1 binding partner, although our results indicate that other relevant binding partners must exist. On the other hand, the expression of CAV1 in melanoma cells is shown to enhance beta1 and alpha5 surface expression, both necessary for adhesion of melanomas to EA.hy926 vascular endothelial cells, possibly via interactions with CAMs (not shown). Furthermore, phosphorylation on Y14 and the surface expression of beta1 integrin are depicted as critical elements for CAV1-enhanced TEM. The presence of CAV1 enhances melanoma adhesion to the endothelium (1 and 2 in TEM scheme) and favors extravasation (3), as well as further colonization into the lung matrix (lung colonization). Taken together, these observations provide a rationale to explain CAV1-enhanced lung metastasis of B16F10 cells.

Finally, our main findings are summarized in a model (Figure 8). Specific cell interactions with fibronectin and laminin are shown to stimulate phosphorylation of CAV1 on Y14 and thereby enhance velocity, persistency and directionality of B16F10 melanoma migration. CAV1 expression is further shown to enhance invasion and FA dynamics in a Y14-dependent manner. Additionally, CAV1 is depicted as increasing alpha5 and beta1 integrin surface expression (left panel, melanoma cell). Both integrins are involved in adhesion of melanomas to vascular endothelial monolayers (see right panel, transendothelial migration), possibly via interactions with cell adhesion molecules (not shown). In the absence of CAV1 both integrins are essentially not present at the cell surface (1). When CAV1 is expressed the integrin heterodimers present at the cell surface favor adhesion (2) and TEM (3). Importantly, only beta1 integrin is essential for CAV1 Y14-driven TEM, while alpha5 integrin is dispensable and can presumably be replaced by other alpha integrin subunits that remain to be defined. Finally, transmigration may also be favored by haptotactic contact with fibronectin/laminin of the lung matrix. Once within the lung matrix rich in the ECM proteins fibronectin and laminin, CAV1 expression in melanoma cells is likely to also favor tissue invasion and colonization (see right panel, lung colonization).

In summary, the CAV1 residue Y14 is shown here to be essential to promote experimental melanoma metastasis to the lung, while not being required for tumor suppression, indicating that the dual role of CAV1 in cancer is attributable to functionally independent regions of the protein. Phosphorylation of CAV1 on Y14 stimulated by binding to fibronectin and laminin, correlated with enhanced migration of B16F10 melanoma cells on these surfaces. Furthermore, CAV1-enhanced trans-endothelial migration required CAV1-phosphorylation on Y14 and beta1 integrin availability on the cell surface. Thus, elevated CAV1 expression in metastatic melanomas is linked here to enhanced beta1 integrin surface expression, trans-endothelial migration and lung metastasis.

## MATERIALS AND METHODS

### Antibodies and reagents

Rabbit polyclonal anti-caveolin-1 (Transduction Laboratories, Lexington, KY), anti-cavin-1 (Sigma-Aldrich, St. Louis, MO), anti-actin (R&D Systems, Minneapolis, MN), anti-integrin beta1 (Santa Cruz Biotechnology, Santa Cruz, CA), anti-integrin beta3 (Santa Cruz Biotechnology, Santa Cruz, CA), anti-HSP90 (Santa Cruz Biotechnology, Santa Cruz, CA) and anti-integrin alpha5 (Santa Cruz Biotechnology, Santa Cruz, CA) antibodies, as well as the mouse monoclonal anti-pY14-caveolin-1 (Transduction Laboratories, Lexington, KY), anti-vinculin (Transduction Laboratories, Lexington, KY), anti-FAK (Santa Cruz Biotechnology, Santa Cruz, CA), anti-pY397-FAK (Cell Signaling Technology), anti-integrin beta3 (FITC) (Abcam, Cambridge, UK) and anti-integrin alphav (Transduction Laboratories, Lexington, KY) antibodies were used as indicated by the manufacturers. Goat anti-rabbit and goat anti-mouse IgG antibodies coupled to horseradish peroxidase (HRP) were from Merck-Millipore (Billerica, Massachusetts, USA) and KPL Laboratories (Washington DC), respectively. Alexa Fluor 488 goat anti-mouse IgG, Alexa Fluor 546 goat anti-rabbit IgG, Alexa Fluor 488 goat anti-rabbit IgG and Alexa Fluor 660 Phalloidin were from Molecular Probes (Invitrogen). DAPI was from Sigma-Aldrich (St. Louis, MO, USA). The ECL chemiluminescent substrate and the BCA protein determination kit were from Pierce (Rockford, IL). The Plasmid Midi Kit was from Qiagen (Valencia, CA). The PCR-Script Amp Cloning kit was from Agilent Technologies (Santa Clara, CA). Bovine collagen-I and mouse laminin were from Invitrogen (Carlsbad, CA). Collagen IV from human placenta and vitronectin were from Sigma-Aldrich (St. Louis, MO, USA). Human fibronectin was from Becton Dickinson (San Jose, CA, USA). Hygromycin was from Calbiochem (La Jolla, CA). Fetal bovine serum (FBS) was from Biological Industries. Cell culture media and antibiotics were from GIBCO (Invitrogen).

### Cell culture

Metastatic murine melanoma cells B16F10 (ATCC, #CRL6475, provided by Laurence Zitvogel, Institut Gustav Roussy, Villejuif, France) were maintained in RPMI 1640 medium supplemented with 10% FBS and antibiotics (100 U/mL penicillin and 100 mg/mL streptomycin). Cells were cultured at 37°C and 5% CO_2_. EA.hy926 endothelial cells (ATCC, #CRL2922, kindly donated by Gareth Owen, Pontificia Universidad Catolica de Chile) were maintained in IMEM medium supplemented with 10% FBS and antibiotics as mentioned above.

### Site directed mutagenesis of Caveolin-1

The Y14F and Y14E mutations were introduced by double PCR, using the primers 5′-cct ctttaccgttcccatcc-3′ (sense) and 5′-gaacggtaaagaggtgccc-3′ (antisense); 5′-gggcacctcgagaccgttccc-3′ (sense) and 5′-catgggaacggtctcgaggtg-3′ (antisense), respectively. Primers were designed to include sequence overlap in the region encompassing the codon for tyrosine 14. External primers used to amplify the full-length caveolin-1 sequence were: 5′-ccgagcgcggccgccatgtctgggggcaaatac-3′ (sense) and 5′-tatctggcggccgcttatgtttctttctgcatgttg-3′ (antisense), both harboring NotI sites. After a double PCR reaction, the final PCR product was cloned into pPCR-Script amp+. Positive colonies were identified and sequenced in both directions. The CAV1-encoding sequences with the Y14F and Y14E mutations were then sub-cloned from pPCR-Script amp+ into the multiple cloning site of pLacIOP, following digestion with NotI. Correct orientation of the insert was determined by PCR using an external anti-sense primer targeting the vector (5′-ttgtctccttccgtgtttca-3′), in combination with the sense primer used to generate the Y14F and Y14E mutations.

### Transfection of B16F10 melanoma cells

The plasmids pLacIOP (referred to as mock) and pLacIOP-caveolin-1 (referred to as CAV1, containing the full-length dog caveolin-1 sequence, NCBI Reference Sequence: NP_001003296.1) were previously described [42, 90]. B16F10 cells were grown to 50-60% confluence in 6 multi-well plates and then transfected with 4 μg of pLacIOP-CAV1(Y14F) (referred as CAV1/Y14F) and pLacIOP-CAV1(Y14E) (referred as CAV1/Y14E), using the FuGene HD reagent, according to the manufacturer's indications. After transfection, cells were plated in complete RPMI medium containing hygromycin (750 μg/mL) for 2 to 3 weeks, to yield stably transfected B16F10(CAV1/Y14F) and B16F10(CAV1/Y14E) cells, respectively.

### Western blotting

Cells grown to 80% confluence were washed twice with cold PBS and lysed in 0.2 mM HEPES (pH 7.4) buffer containing 0.1% SDS, phosphatase inhibitors (1 mM Na_3_VO_4_) and a protease inhibitor cocktail (10 mg/mL benzamidine, 2 mg/mL antipain, 1 mg/mL leupeptin, 1 mM PMSF). Protein concentrations were determined using the BCA assay. Total protein extracts (30 μg/lane) were separated by SDS-polyacrylamide gel electrophoresis (SDS-PAGE) and then transferred to a nitrocellulose membrane. Blots were blocked with 5% milk in 0.1% Tween-PBS and then probed with anti-actin (1:5000), anti- caveolin-1 (1:5000), anti-integrin beta1 (1:1000), anti-integrin beta3 (1:1000), anti-integrin alphav (1:3000) anti-integrin alpha5 (1:1000) and anti-HSP90 (1:3000) antibodies. Alternatively, blots were blocked with 5% gelatin in 0.1% Tween-PBS for incubations with anti-pY14-caveolin-1 (1:3000) antibody. Bound antibodies were detected with HRP-conjugated secondary antibodies and the ECL system.

### Multi-wounding assay

Cells were grown to confluency for 24 h in complete medium and then serum starved for 60 min. Subsequently, monolayers were multiply wounded with a steel comb and remaining cells were allowed to migrate into the available space on plates with medium containing 3% serum for 5, 15, 30, 45 and 60 min. Whole cell lysates were prepared and pY14-CAV1 levels were determined by Western Blotting.

### Transwell migration assay

Assays were performed in Boyden Chambers (Transwell Costar, 6.5 mm diameter, 8 μm pore size) according to the manufacturer's instructions. Briefly, the lower surfaces of the inserts were coated with 2 μg/ml fibronectin. B16F10 cells (5×10^4^) re-suspended in serum-free medium were plated in the top of each chamber insert and serum-free medium was added to the bottom chamber. After 2 h, inserts were removed, washed and cells that migrated to the lower side of the insert membranes were stained with 0.1% crystal violet in 2% ethanol and counted using an inverted microscope.

### Invasion assay

Assays were performed in matrigel chambers (matrigel BD, 6.5 mm diameter, 8 μm pore size) according to the manufacturer's instructions. Cells (5×10^4^) re-suspended in serum-free medium were plated on top of each chamber insert and complete medium was added to the bottom chamber. After 22 h, inserts were removed, washed and cells that migrated to the lower side of the insert membranes were stained with toluidin blue in 1% methanol and counted using an inverted microscope.

### Microfluidic multichannel migration device

Microfluidic migration devices allow the simultaneous analysis of multiple repeats of cell migration into the microchannels covered with defined ECM surfaces, as described previously [56]. The most important feature of these devices is that a confluent cell monolayer is established within the main compartment without any cell migration into the microchannels, until they are filled with culture medium, which allows the maintenance of a virgin surface within the migrating channels. Channels were constructed and assayed previously [56], and the glass surfaces, which are the base of the microchannels, were functionalized by allowing extracellular matrix (ECM) molecules to adsorb onto the glass substrate overnight at 4°C. The ECM molecules (fibronectin, laminin, collagen I, collagen IV and vitronectin) were solubilized (50 μg/ml) in phosphate buffered saline (PBS). Migration was recorded by time-lapse microscopy for 7 h using a spinning disk confocal microscope (IX81, Olympus) and a 12-BIT CCD camera (XM10, Olympus). Instant velocity refers to the velocity of migration of each cell at any given time point. Average velocity is calculated as the net distance of migration (Euclidian distance) divided by the time required to cover that distance for any given cell. Cell persistency was quantified as the ratio of the net distance divided by the total distance of movement (ID) for each cell. Directionality of cell migration (cell orientation) was evaluated with the Image J Software (plugin “chemotaxis”) by placing cell tracks in a Cartesian coordinate system. Cell tracks that remained within a 60°C angle with respect to the direction of cell movement were considered as directional.

### Preparation of focal adhesion-enriched fractions

Fractionation was performed as described previously [91]. In brief, cells were plated onto fibronectin-coated plates (2 μg/ml) and allowed to attach for 45 min. Adherent cells were pre-extracted with lysis buffer containing 0.5% Triton X-100 at 4°C for 30 min; this fraction is referred to as “cell fraction”. The remaining fraction attached to the plate was extracted with RIPA buffer for 5 min on ice and scraped off the plates. Fractions were clarified by centrifugation at 14000g for 10 min. This fraction is referred to as “focal adhesion-enriched fraction”. Both cell and focal adhesion fractions were analyzed by Western blotting.

### Cell adhesion assay

Cells (2,5×10^4^) were suspended in serum-free medium and allowed to attach to the ECM molecules coated-96 well plates (2 μg/ml) for different periods of time, as indicated. Non-adherent cells were removed by washing gently in serum-free medium and adherent cells were stained with 0.1% crystal violet in 2% ethanol. Cell-bound dye was eluted with methanol and the absorbance was measured at 590 nm.

### Spreading assay

Cells were suspended in serum-free medium and allowed to attach to the plates coated with different ECM molecules (2 μg/ml) for the indicated periods of time in each experiment. The area per cell (μm^2^) and levels of pY14-CAV1 were analyzed by epifluorescence microscopy (Spinning-disc microscope IX81, Olympus) and Western Blotting, respectively.

### Wound-healing migration assay

Confluent monolayers of B16F10 cells were wounded with a 20-200 μl pipette tip. Cells were washed twice with PBS and anti-integrin beta1 (5 μg/ml), anti-integrin beta3 (5 μg/ml) or anti-integrin alpha5 (5 μg/ml), suspended in serum-free medium, were added. Image series were acquired using a 10X objective lens in an inverted microscope (Olympus CKX41) using a digital sight DS-2MBWc Nikon camera. Cells were allowed to migrate for 7 h and migration was quantified as the percentage of wound closure using the Adobe Photoshop C3 software (Adobe Systems, San Jose, CA).

### Flow cytometry

Cells were detached using trypsin/EDTA and incubated at 4°C to avoid internalization of surface proteins. After blocking with BSA 2%, cells were immunolabeled with primary antibody anti-integrin beta1 (1:25) or anti-integrin alpha 5 (1:25). Cells were then washed and incubated with the secondary antibody anti-goat Alexa 488 (1:200). Alternatively, cells were immunolabeled with anti-integrin beta 3 (FITC) (1:50) for 30 min at 4°C. Cells were analyzed using a FACS Canto (BD Bioscience) flow cytometer.

### Focal adhesion assembly and disassembly assay

To evaluate FA assembly and disassembly, B16F10 cells were transiently transfected with the plasmid encoding vinculin-EGFP (pEGFP-vinculin, kindly donated by Kris DeMali, University of Iowa [92]. After transfection (24 h), cells were re-plated onto chambered coverglass slips (Nunc, Lab-Tek II, ThermoScientific), pre-coated with fibronectin (2 μg/ml), allowed to spread in serum-free medium and then stimulated (pulsed) with 3% FBS. Cells were visualized using a confocal microscope (FluoView FV10i, Olympus) coupled to a carbon dioxide maintenance device. Images were captured at time intervals of 2 min for 1 h. For FA analysis, these structures were defined in terms of function and size with the Image J Software (Urra et al., 2012). The time of FA formation was taken from the pulse of serum until these structures were well defined, as described previously [42]. The time required for FA disassembly was defined as the time from when these structures begin to disappear until they disappeared completely.

### Analysis of CAV1 distribution

To evaluate CAV1 distribution, B16F10 cells were plated on coverslips pre-coated with fibronectin (2 μg/ml), allowed to attach in serum-free medium and then stimulated for 15, 30 and 45 min with 3% FBS. Cells were fixed at the indicated time points with 4% paraformaldehyde in 100 mM PIPES buffer pH 6.8, containing 40 mM KOH, 2 mM EGTA and 2 mM MgCl2 for 30 min. After washing (3 times with 50 mM Tris buffer pH 7.6 containing 0.15N NaCl and 0.1% sodium azide), cells were permeabilized with 0.1% Triton X-100 in washing solution for 10 min, washed twice and then blocked with 1% bovine serum albumin for 60 min. CAV1 distribution was evaluated by staining cells with an anti-CAV1 pAb (1:200) and FAs were stained with anti-vinculin mAb (1:200), followed by Alexa Fluor 546 anti-rabbit IgG (1:200) and Alexa Fluor 488 anti-mouse IgG (1:400). DAPI (0.5 mg/mL) was used for nuclear staining. Coverslips were washed and mounted on microscope slides with 10% Mowiol-2.5% 1,4-Diazabicyclo [2.2.2] octane and samples were visualized with a Nikon Spectral C2 Plus microscope (pixel 80 nm). Samples were analyzed with the Fiji Software (http://fiji.sc/). Total fluorescence and ROI (Region of Interest, corresponding to the peripheral, sub-plasma membrane fluorescence) were quantified after 30 and 45 min of spreading. Fluorescence was calculated as Integrated Density - (Area of selected cell * Mean fluorescence of background readings). Distribution of CAV1 in the cell periphery is shown in percent (%) and was calculated as (border fluorescence*100)/whole cell fluorescence). To analyze the area and number of FAs per cell, threshold was applied in selected FAs and the plug-in “particle analysis” using the Fiji Software was applied. For CAV1 distribution in FAs, the “RGB profiles” mode of Fiji was employed. Line scans were drawn from the cell membrane across FAs. Fluorescence intensity and distance (in pixels) of these line scans were calculated to generate distribution profiles.

### Tumor formation assay

B16F10 cells (3 × 10^5^) suspended in 100 μL of physiological saline solution (0.9% NaCl) were injected subcutaneously into the right flank of mice. Appearance of tumors was monitored by palpation. The largest perpendicular diameters of the resulting tumors were periodically measured, and tumor volumes were calculated according to the following formula: width x length x π/6 (Current Protocols in Immunology, 2000). Animals were sacrificed when tumors reached the bioethically permitted limit of 2500 mm^3^ [28].

### Metastasis assay

B16F10 cells (2 × 10^5^), suspended in 500 μL of physiological saline solution, were injected intravenously into the tail vein of C57BL/6 mice. The animals were sacrificed 21 d post-injection. Lungs were fixed in Feketes solution [28] and black metastatic tissue from lung was weighed. Metastasis was expressed as black tissue mass/total lung mass in percent (%) after fixation of the tissue [28]. Note that for tumor formation and metastasis assays male and female mice were used indifferently.

This study was performed according to the rules and standards established by the Bioethics Committee on Animal Research at the Facultad de Medicina, Universidad de Chile (Protocol number CBA # 0416 FMUCH).

### Transendothelial migration assay (TEM) and adhesion assays

EA.hy926 cells (2.5 × 10^5^) were grown to confluency (after 72 h) on top of an 8-μm-pore size membrane (Transwells; BD Biosciences) [93] for TEM and over coverslips for adhesion assays. B16F10 cells (5 × 10^4^) were labeled with CellTracker Green (5 μM; Life Technologies), incubated with neutralizing anti-integrin beta1 and alpha5 antibodies (anti-HA antibody as control) and added to the top of the transwell inserts for TEM or added on endothelial EA.hy926 cells for adhesion assays. After 6 h, transwells were washed with PBS and wiped with cotton swabs. Inserts and coverslips were fixed with 4% para-formaldehyde in PBS, stained with DAPI and mounted onto glass slides. Migrated green labeled-B16F10 cells were imaged by epifluorescence microscopy (IX81, Olympus).

### Transmission electron microscopy

B16F10(mock), B16F10(CAV1/wt) and NIH-3T3 (positive control) cells were prepared for electron microscopy following standard protocols. Briefly, cells suspended in PBS were pelleted, washed twice with PBS and post-fixed in 1% osmium tetroxide in PBS pH 7.4 for 2 h, then washed again three times in PBS and dehydrated in an increasing concentration series of acetone on ice. Cells were infiltrated in 1:1 acetone/EPON for 2 h at room temperature and finally embedded in fresh resin. Thin sections (60–80 nm) were cut with a diamond knife (Diatome, Washington D.C., USA) using a Leica ULTRACUT R ultramicrotome and collected on 200-mesh copper grids. The sections were stained with saturated uranyl acetate in methanol and lead citrate, observed under a Zeiss 900 electron microscope at 80 kV and photographed with a Gatan Orius SC1000 - 832, CCD camera. All electron microscopy supplies were from Pelco (Ted Pella, Inc., Redding, CA).

### Statistical analysis

Results were statistically compared using the Kruskal-Wallis ANOVA test followed by multiple comparison post-tests (Dunn's multiple comparison test). For paired groups, the Mann-Whitney test was employed. Data analyzed in this manner are specifically indicated in the respective Figure legends. All groups were obtained from three or more independent experiments. *p <0.05 was considered significant.

## SUPPLEMENTARY FIGURES


