# Supplementary material for: Extracellular matrix-specific Caveolin-1 phosphorylation on tyrosine 14 is linked to augmented melanoma metastasis but not tumorigenesis
Source: Oncotarget. 2016 May 31;7(26):40571–93. doi: 10.18632/oncotarget.9738 (PMC5130029; doi:10.18632/oncotarget.9738)
Supplement: Supplementary file 1 [file oncotarget-07-40571-s001.pdf]

Extracellular matrix-specific Caveolin-1 phosphorylation on tyrosine 14 is linked to augmented melanoma metastasis but not tumorigenesis

SUPPLEMENTARY FIGURES

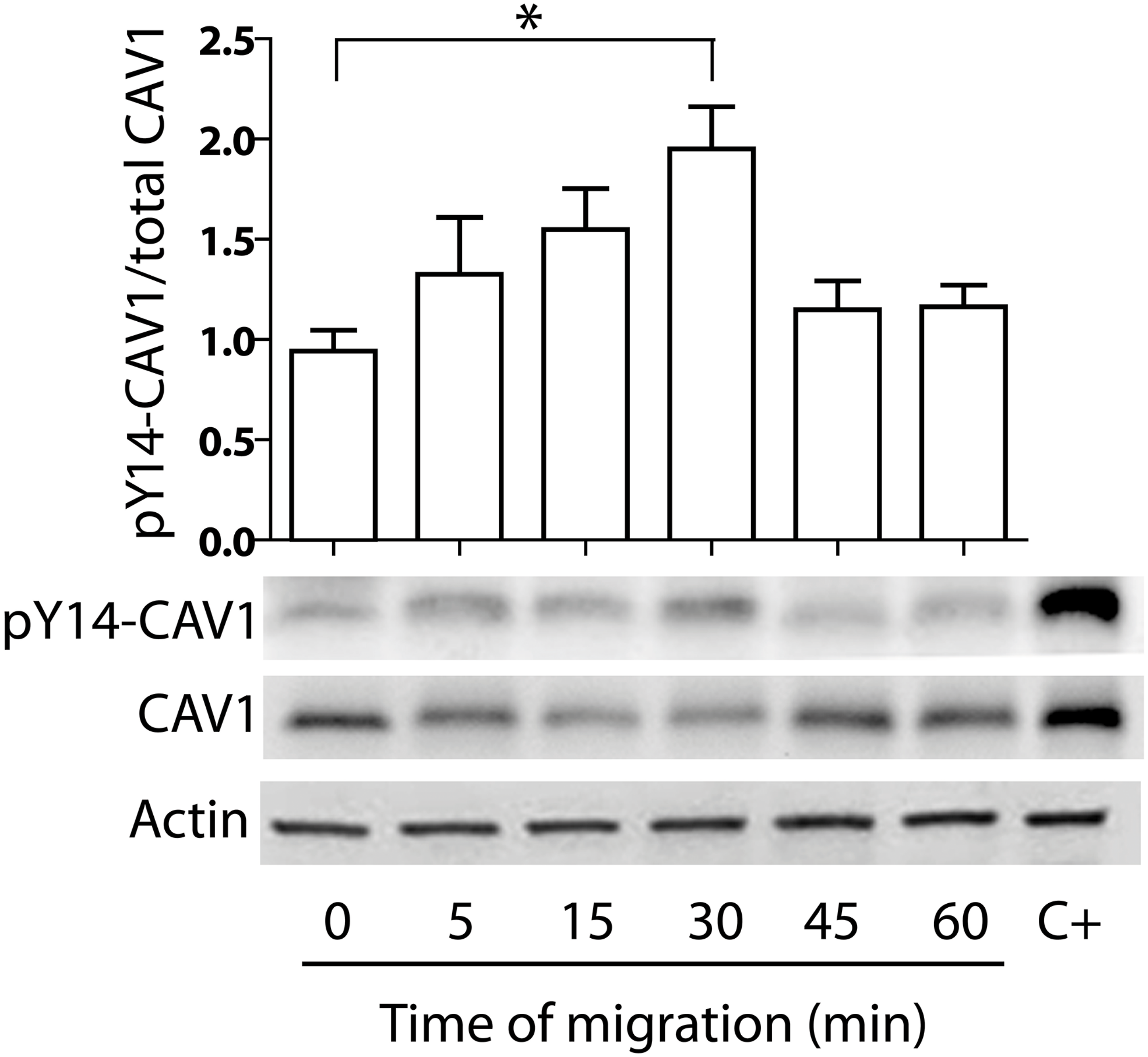

Supplementary Figure S1: Phosphorylation of CAV1 upon multiple wounding of a cell monolayer. B16F10(CAV1/wt) cells were grown to confluency and monolayers were multiply wounded with a steel comb. Cells were allowed to migrate for different periods of time (0, 15, 30, 45 and 60 min) and whole cell lysates were prepared. pY14-CAV1 levels were determined by Western Blotting. *Upper graph*, relative pY14-CAV1 levels normalized to total CAV1 by scanning densitometry are shown as the fold-increase with respect to the "0" time point (before scratch). Data represent the average of three independent experiments (mean ± S.E.). Statistically significant differences are shown (*$p<0.05$). *Lower panels,* representative Western Blot images of results from three independent experiments. As a positive control (C+) for pY14-CAV1, cells were treated with 5 mM H_2O_2 for 20 min.

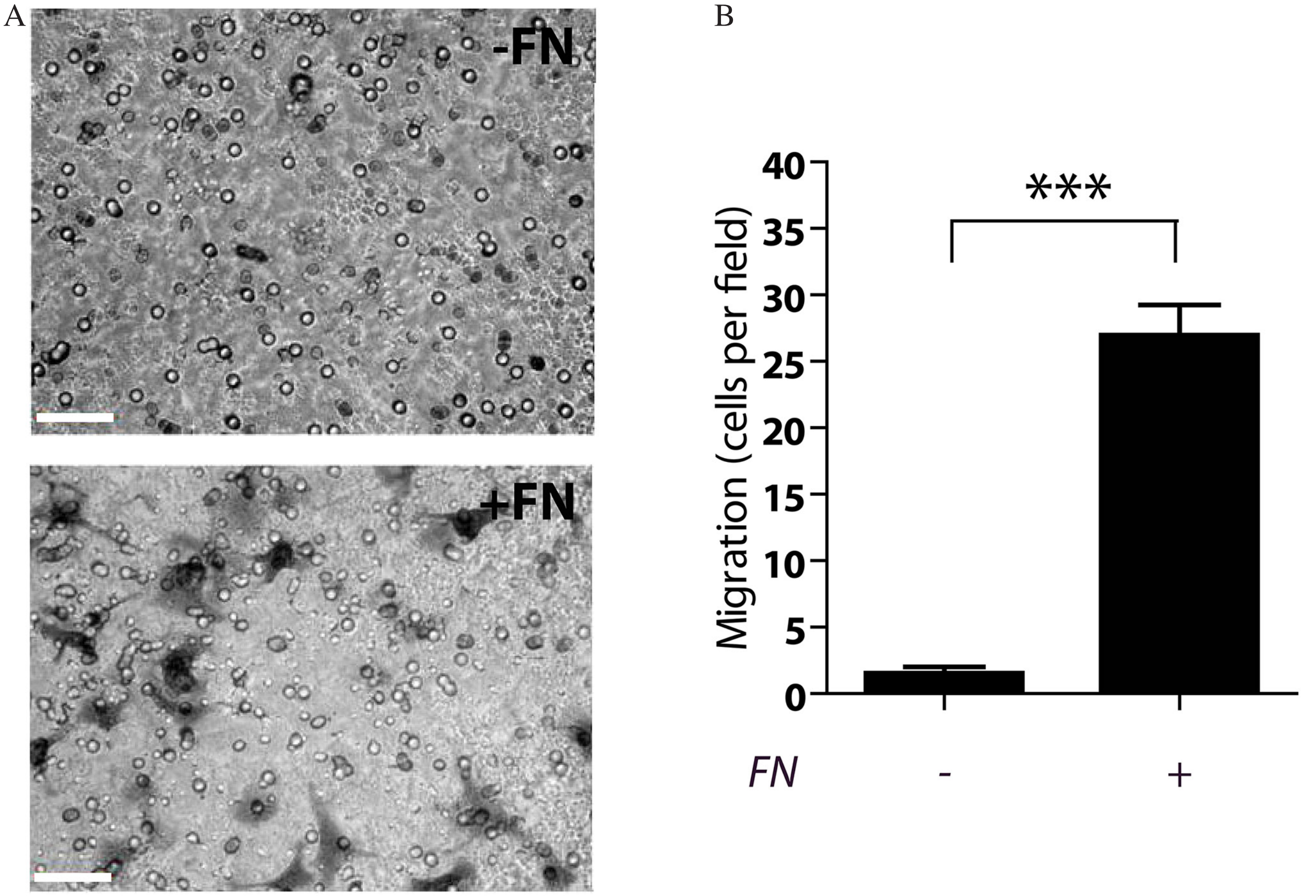

Supplementary Figure S2: Effect of fibronectin on transmigration of B16F10 (mock) cells. B16F10(mock) cells (5x10^4) were added to Transwell inserts pre-coated or not on the lower side with fibronectin (2 µg/ml). **A.** Cells were allowed to migrate for 2 h and then detected after fixation on the lower side of the membrane by crystal violet staining. Images of the transwell inserts observed at 400X magnification are shown (scale bar, 100 µm). **B.** Data averaged from 6 different fields in three independent experiments were normalized to values obtained for B16F10(mock) cells (mean ± S.E.M, ***p<0.001).

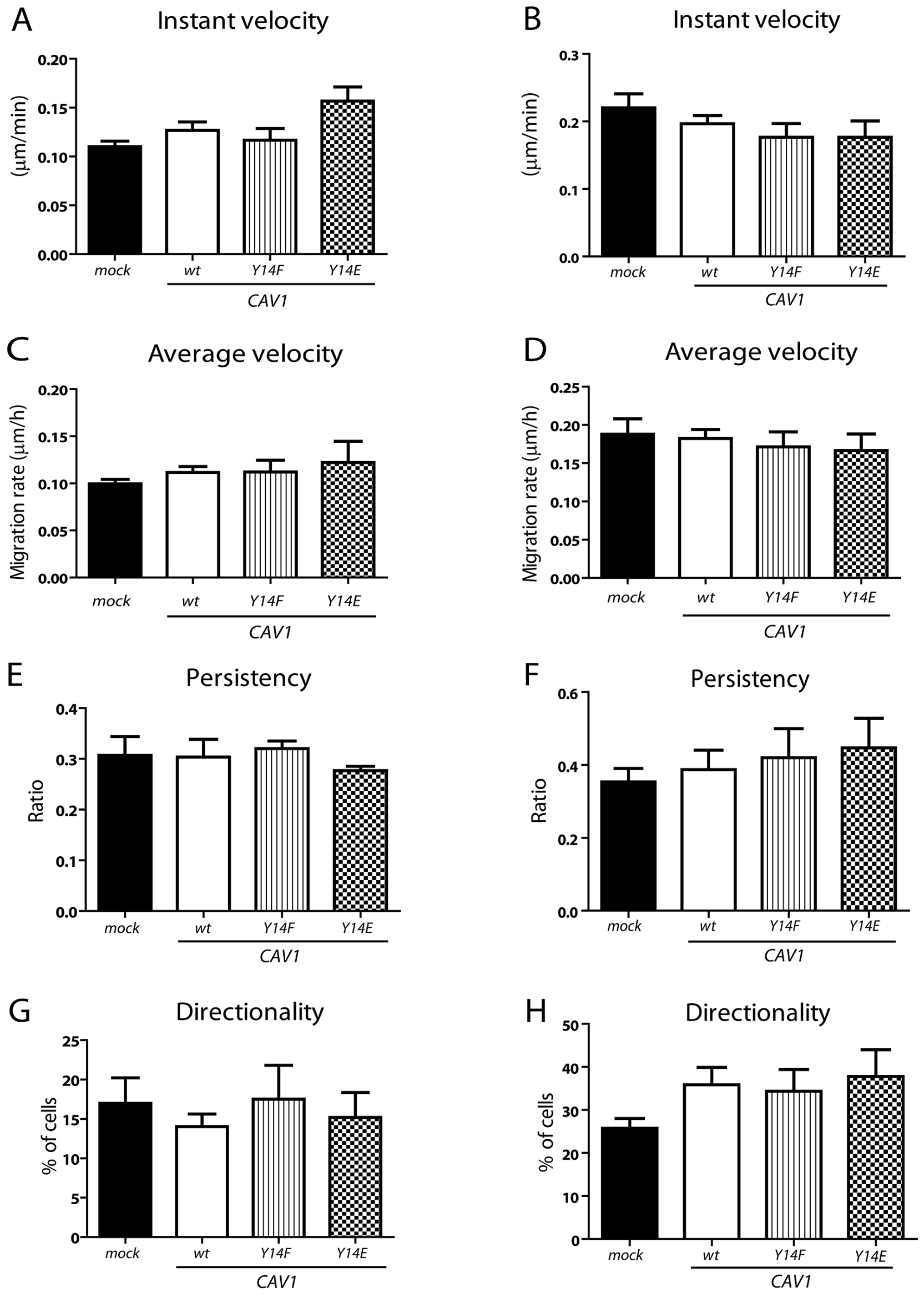

Supplementary Figure S3: Migration of B16F10 melanoma cells on collagen IV and vitronectin. B16F10(mock), (CAV1/wt), (CAV1/Y14F) and (CAV1/Y14E) cells were induced with IPTG 1 mM for 48 h. Then, 1x10^6 cells were seeded in the central chamber of the migration micro-device, pre-coated in the side-channels with collagen IV or vitronectin (50 µg/ml). Cells were allowed to attach for 2 h and migration was recorded by time-lapse video microscopy for 7 h (15-min time intervals). Cell tracks were determined using the Image J Software ("Manual Tracking" plug-in). **A–B.** Instant velocity (µm/min) at any given time point was analyzed for individual cells during tracking on collagen IV and vitronectin, respectively. **C–D.** TheAverage velocity was obtained as the quotient between the Euclidean distance (µm) and the total time of migration while tracking the cells on collagen IV and vitronectin, respectively. **E–F.** Persistency of migration was calculated as the ratio between the net distance and the total distance of migration on collagen IV and vitronectin, respectively. **G–H.** Directionality of migration (% of cells) was determined, whereby cell tracks within a 60° angle with respect to the direction of cell movement were considered as oriented during migration on collagen IV and vitronectin, respectively. Each graph shows the values for each parameter averaged from three independent experiments (mean ± S.E.M, n = 3).

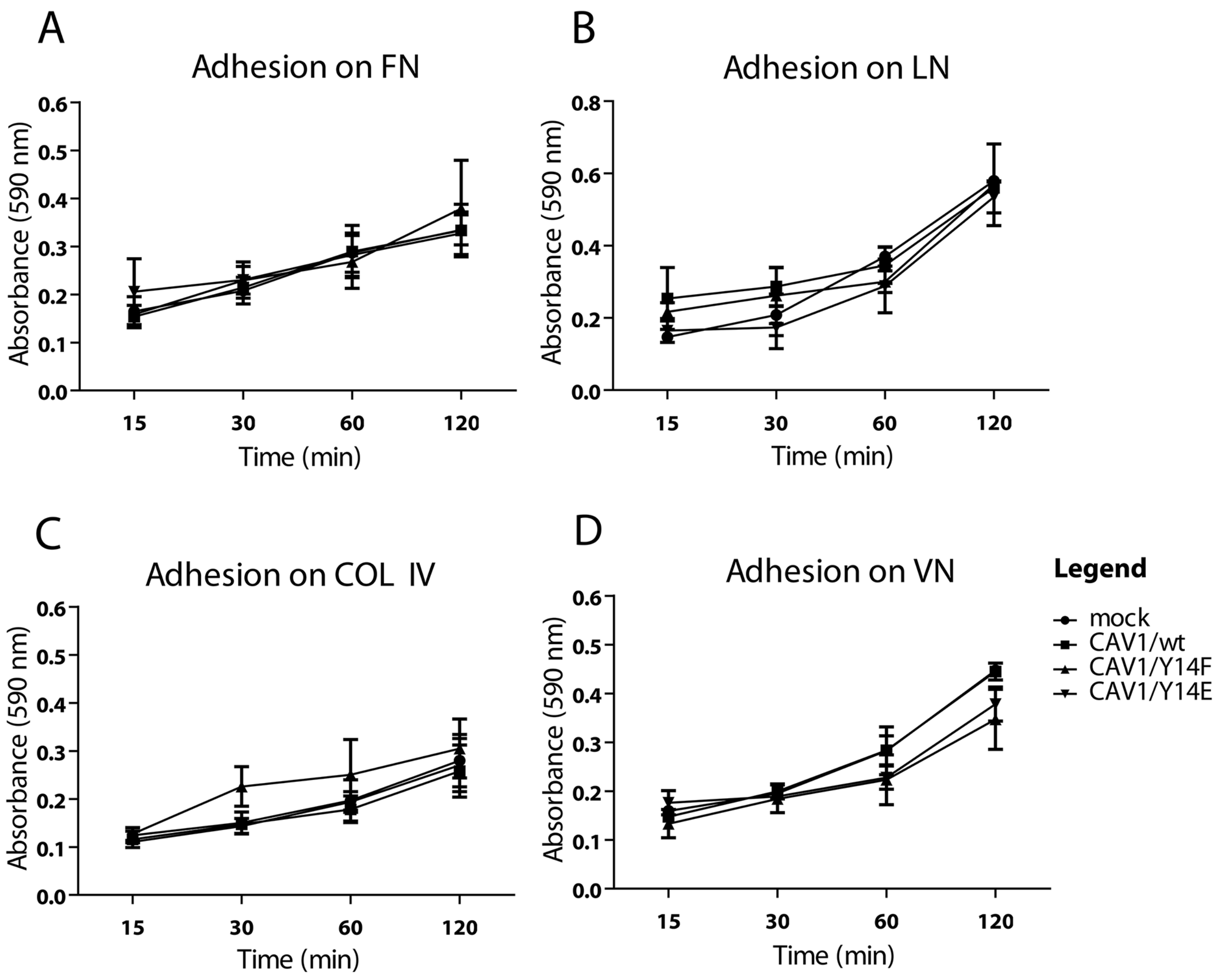

Supplementary Figure S4: Adhesion of B16F10 cells to pure ECM surfaces. B16F10(mock), (CAV1/wt), (CAV1/Y14F) and (CAV1/Y14E) were induced with IPTG 1 mM for 48 h. In adhesion assays, cells (2,5x10[4]) were allowed to attach to **A.** fibronectin-, **B.** laminin-, **C.** vitronectin- and **D.** collagen IV-coated plates (2 µg/ml) for different periods of time (15, 30, 60 and 120 min). Adherent cells were stained with 0.1% crystal violet and the absorbance was measured at 590 nm. Data represent the averages from three independent experiments (mean ± S.E.M, n=3).

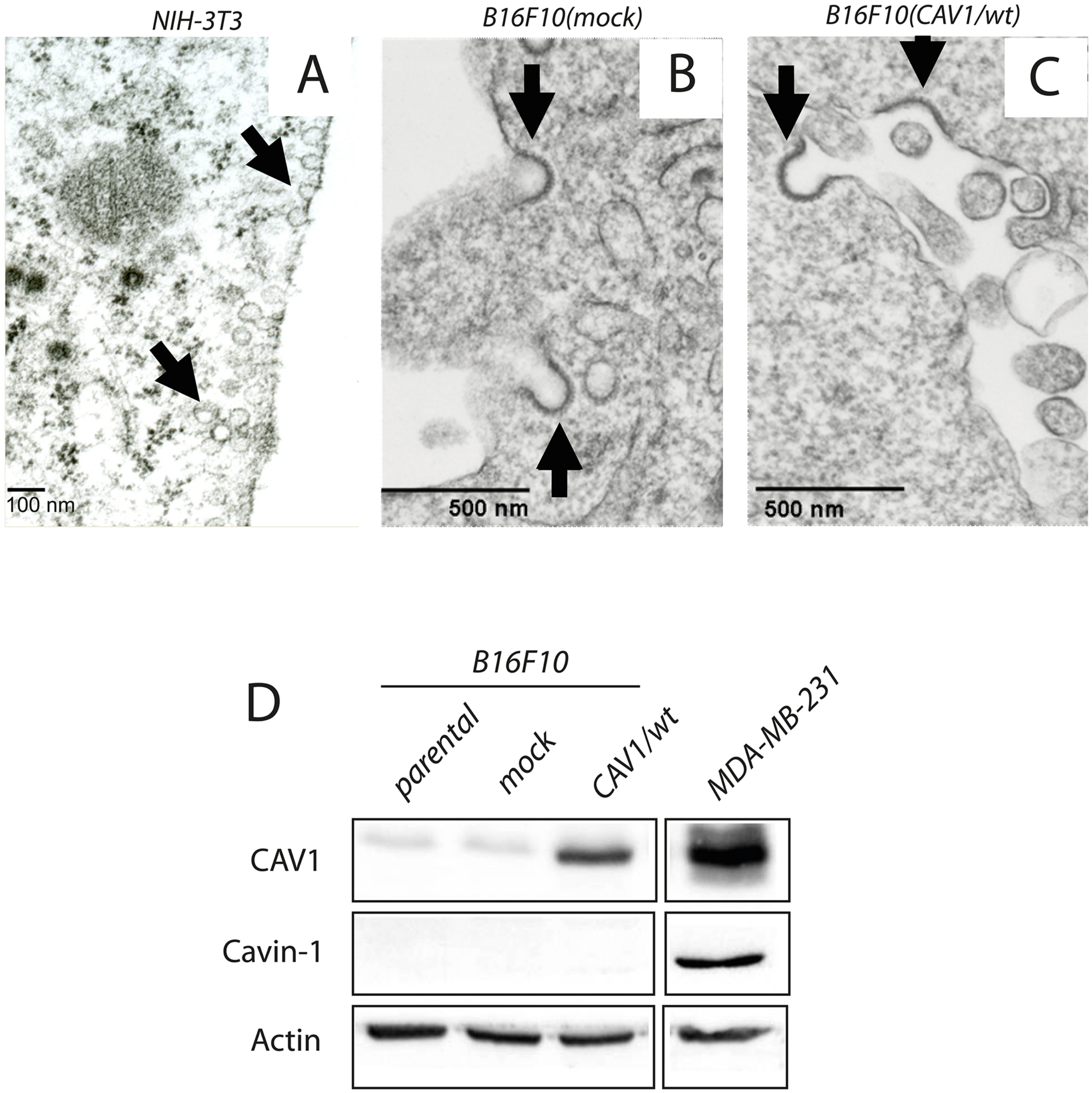

Supplementary Figure S5: Caveolae presence and Cavin-1 expression in B16F10 cells. B16F10(mock), B16F10(CAV1/wt) and NIH-3T3 (positive control) cells were fixed and treated for electron microscopy analysis as decribed in the Materials and Methods section. **A.** NIH-3T3 cell are shown at 11500x magnification (scale bar, 100 nm). Black arrows indicate caveolae in the image. **B.** B16F10(mock) and **C.** B16F10(CAV1/wt) cells are shown at at 21000x magnification (scale bar, 500 nm). Black arrows indicate coated pits in the images. **D.** Cavin-1 expression was analyzed by Western Blotting in total cell extracts of B16F10 and MDA-MB-231 (positive control) cells. Note that sample lanes shown here were all run in the same gel and transferred to a nitrocellulose membrane. The image was edited to remove irrelevant samples in between the CAV1/wt and MDA-MB-231 lanes. Blots shown for anti-CAV1, anti-Cavin-1 and anti-Actin (control) are representative of results from three independent experiments.

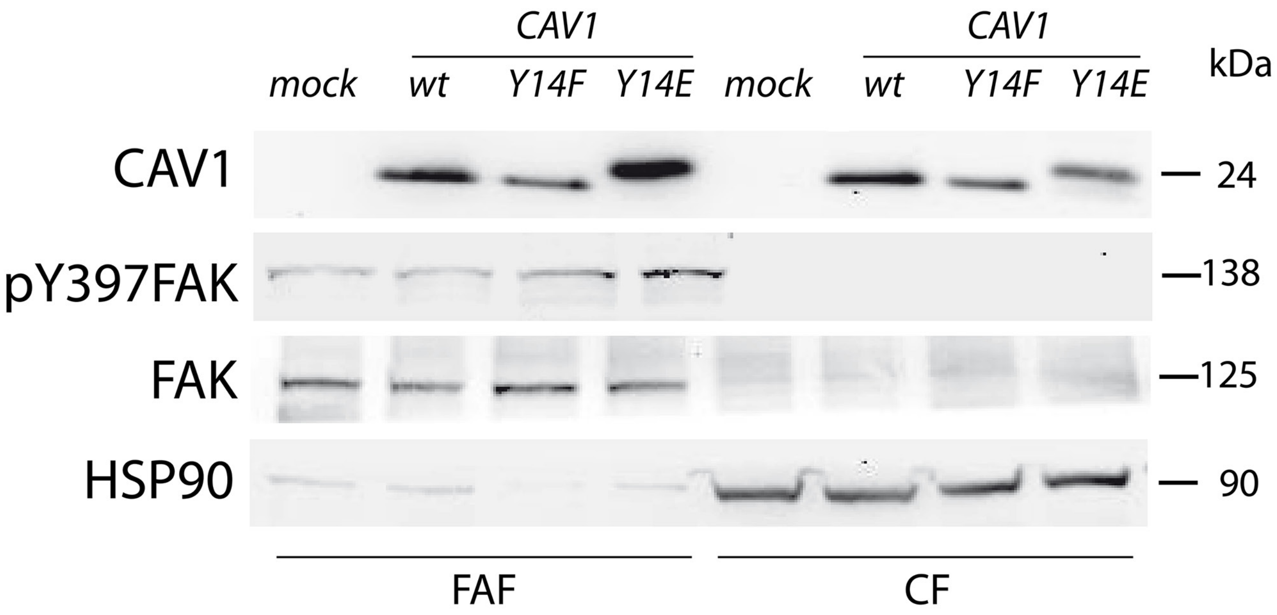

Supplementary Figure S6: CAV1 presence in FA-enriched fractions of B16F10 cells. B16F10(mock), (CAV1/wt), (CAV1/Y14F) and (CAV1/Y14E) cells were induced with IPTG 1 mM for 48 h. Then, 1×10^6 cells were plated onto fibronectin-coated plates (2 µg/ml) and allowed to attach for 45 min. Adherent cells were pre-extracted with lysis buffer to obtain a "cytosolic fraction" (CF). The remaining fraction attached to the plate was extracted and referred to as "focal adhesion-enriched fraction" (FAF). Both cytosol and focal adhesion fractions were analyzed by Western blotting. As a positive control for the FA fraction, FAK and beta1 integrin levels were evaluated. HSP90 levels were evaluated as a negative control. The image is representative of results from three independent experiments.

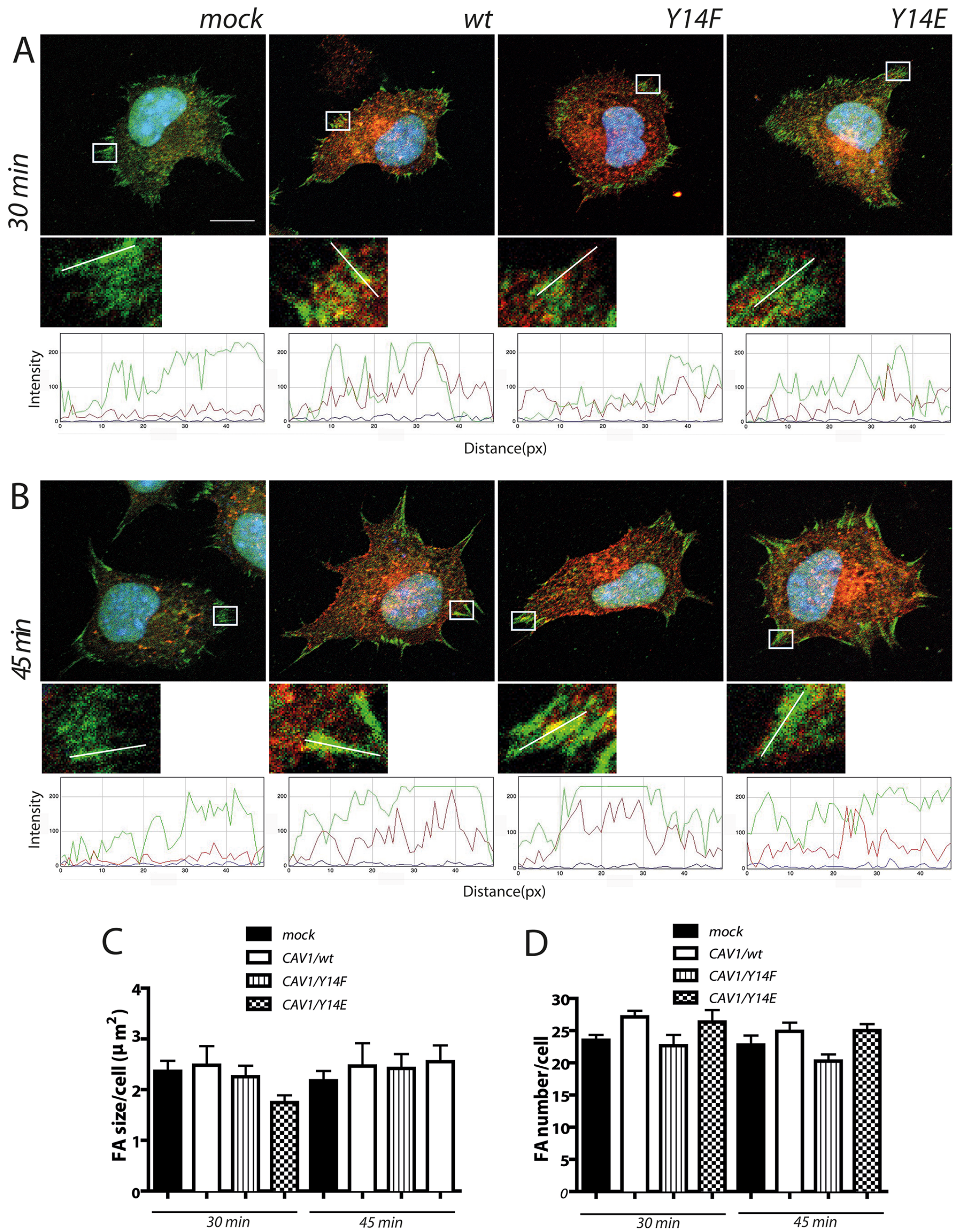

Supplementary Figure S7: CAV1/vinculin co-distribution and FA-size and number in B16F10 cells. B16F10(mock), (CAV1/wt), (CAV1/Y14F) and (CAV1/Y14E) cells were induced with 1mM IPTG for 48 h. Cells were seeded on fibronectin-coated chambered slides (2 µg/ml) and grown in the presence of IPTG (1 mM) for 24 h. Thereafter, cells were serum-starved for 60 min, pulsed with 3% serum and fixed at 15, 30 and 45 min of spreading for CAV1 and vinculin detection by immunofluoresence microscopy. Samples were analyzed with the Fiji Software. For CAV1 distribution in FAs a line (representing an optical section) was drawn from the plasma membrane to the cell interior and intensity of fluorescence and distance (px) were calculated to generate distribution profiles. Area of FAs and number of FAs per cell was analyzed by Fiji Softawe using treshold and "particle analysis" plugins. **A.** Images of the distribution of CAV1 (red) in FAs (green) after 30 min of spreading. Zoomed images of the analyzed area are shown. **B.** Images of the distribution of CAV1 in FAs after 45 min of spreading. Zoomed images of the analyzed area are shown. Note that the histograms show the distribution profiles of CAV1 proteins (red) and vinculin (green) in FAs. **C–D.** Graphs summarizing the average FA-size/cell (µm^2) (C) and the average FA-number/cell (D) for each experimental condition in B16F10 cells after 30 and 45 min of spreading. Note that images of cells shown here are the same as in Figure 5 (panels B and C), but additionally show vinculin as a FA marker to assess co-distribution with CAV1 (insert panels), as well as to quantify FA size and number (panels C and D). Images are representative of results from three independent experiments.

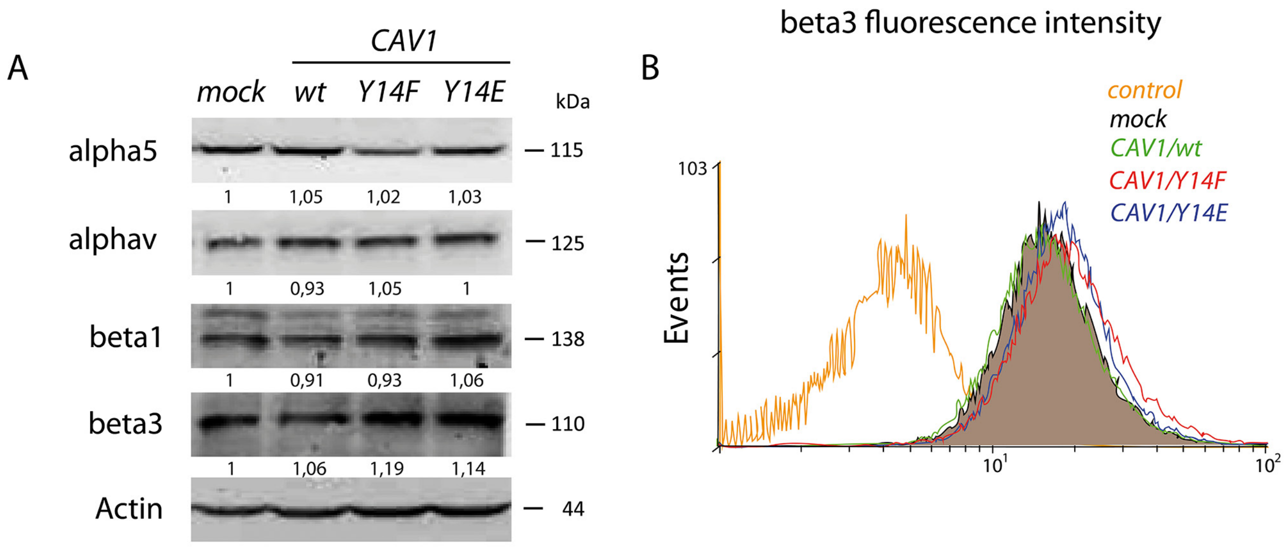

Supplementary Figure S8: Integrin presence and surface expression in B16F10 cells. B16F10(mock), (CAV1/wt), (CAV1/Y14F) and (CAV1/Y14E) cells were induced with IPTG 1 mM for 48 h. **A.** Then, total integrin expression was evaluated by Western Blotting of all extracts (50 µg of protein per lane). Blots shown for anti-alpha5, anti-alphav, anti-beta1, anti-beta3 and anti-Actin (control) are representative of results from three independent experiments. Relative alpha5, alphav, beta1 and beta3 levels normalized to β-Actin by scanning densitometry are shown as the fold-increase with respect to the (mock) condition. Average values for each condition are: Integrin alpha5: mock (1±0,15); wild type CAV1 (1,05±0,18); CAV1/Y14F (1,02±0,12); CAV1/Y14E (1,03±0,12). Integrin alphav: mock (1±0,21); wild type CAV1 (0,93±0,05); CAV1/Y14F (1,05±0,19); CAV1/Y14E (1±0,06). Integrin beta1: mock (1±0,1); wild type CAV1 (0,91±0,1); CAV1/Y14F (0,93±0,06); CAV1/Y14E (1,06±0,15). Integrin beta3: mock (1±0,12); wild type CAV1 (1,06±0,1); CAV1/Y14F (1,19±0,02); CAV1/Y14E (1,14±0,12). **B.** Surface expression of beta3 integrin in B16F10 cells was determined by flow cytometry after incubating fixed cells with anti-beta3 integrin antibody. The histogram of beta3 fluorescence intensity is representative of results from three independent experiments.

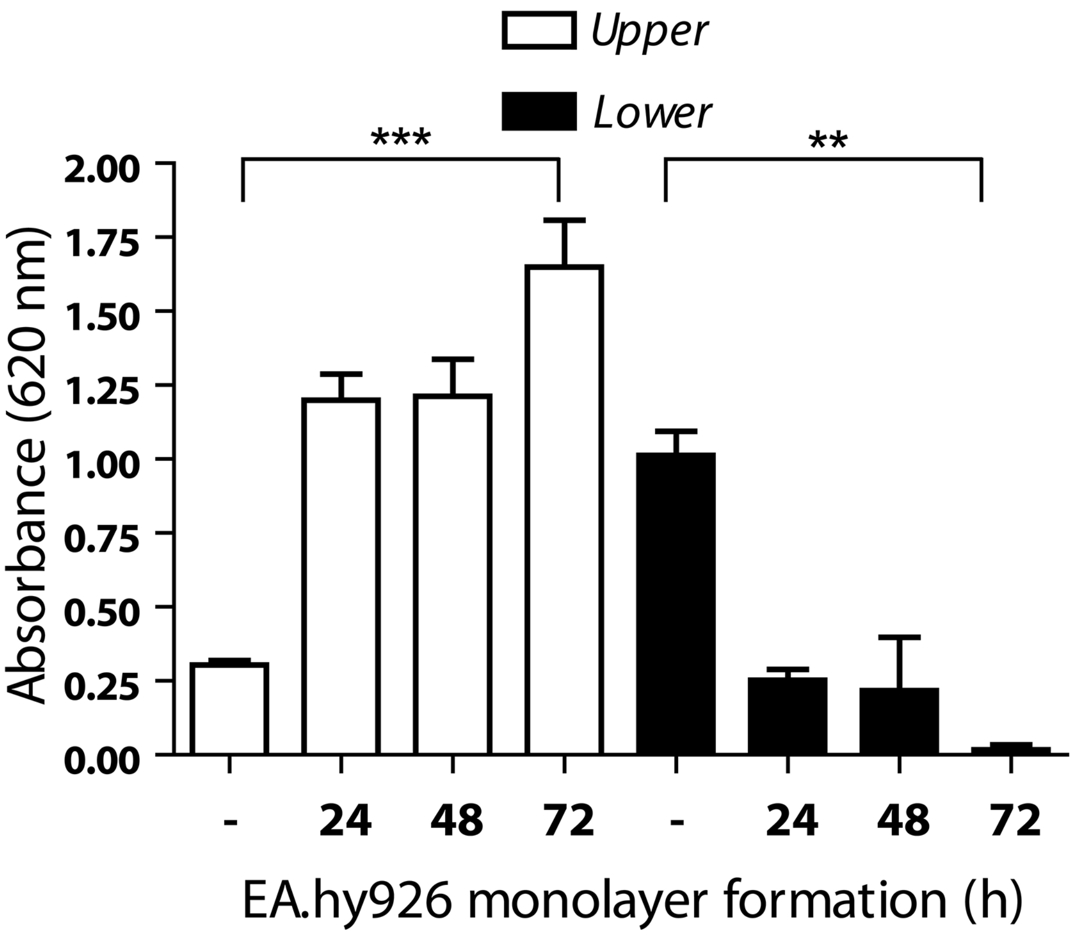

Supplementary Figure S9: Blue dextran permeability of a monolayer of EA.hy926 cells. EA.hy926 cells (2,5 x 10^5) were seeded on Transwell inserts to generate monolayers for 24, 48 and 72 h. Blue-dextran was added to the upper compartment of the insert to evaluate permeability of cell monolayers at the indicated time points. Absorbance (620 nm) of the solutions on both sides of the insert (upper and lower) was measured after 24, 48 and 72 h. White bars indicate values of absorbance in the upper compartment; black bars indicate values of absorbance in the lower compartment.
